# Organophosphate Esters in China: Fate, Occurrence, and Human Exposure

**DOI:** 10.3390/toxics9110310

**Published:** 2021-11-16

**Authors:** Zhihui Hu, Lingshi Yin, Xiaofeng Wen, Changbo Jiang, Yuannan Long, Jiawei Zhang, Ruyi Liu

**Affiliations:** 1 School of Hydraulic Engineering, Changsha University of Science & Technology, Changsha 410114, China; 2059133566@stu.csust.edu.cn (Z.H.); jiangchb@csust.edu.cn (C.J.); lynzhb@csust.edu.cn (Y.L.); zjw@stu.csust.edu.cn (J.Z.); ruyi@stu.csust.edu.cn (R.L.); 2 Key Laboratory of Dongting Lake Aquatic Eco-Environmental Control and Restoration of Hunan Province, Changsha 410114, China; 3 Key Laboratory of Water-Sediment Sciences and Water Disaster Prevention of Hunan Province, Changsha 410114, China; 4 College of Environmental Science and Engineering, Hunan University and Key Laboratory of Environmental Biology and Pollution Control (Hunan University), Ministry of Education, Changsha 410082, China; yin@csust.edu.cn

**Keywords:** China, human exposure, organophosphate esters, sources, spatial distribution

## Abstract

Organophosphate esters (OPEs) are widely used as flame retardants and plasticizers. OPEs have been released into various environments (e.g., water, sediments, dust and air, and soil). To investigate the occurrence and distribution of OPEs in various environments in China, this review collects and discusses the published scientific studies in this field. Chlorinated OPEs, as flame retardants, are the predominant OPEs found in the environment. The analysis of data revealed large concentration variations among microenvironments, including inflowing river water (range: 0.69–10.62 µgL^−1^), sediments (range: 0.0197–0.234 µg/g), dust (range: 8.706–34.872 µg/g), and open recycling sites’ soil (range: 0.122–2.1 µg/g). Moreover, OPEs can be detected in the air and biota. We highlight the overall view regarding environmental levels of OPEs in different matrices as a starting point to monitor trends for China. The levels of OPEs in the water, sediment, dust, and air of China are still low. However, dust samples from electronic waste workshop sites were more contaminated. Human activities, pesticides, electronics, furniture, paint, plastics and textiles, and wastewater plants are the dominant sources of OPEs. Human exposure routes to OPEs mainly include dermal contact, dust ingestion, inhalation, and dietary intake. The low level of ecological risk and risk to human health indicated a limited threat from OPEs. Furthermore, current challenges and perspectives for future studies are prospected. A criteria inventory of OPEs reflecting the levels of OPEs contamination association among different microenvironments, emerging OPEs, and potential impact of OPEs on human health, particularly for children are needed in China for better investigation.

## 1. Introduction

Organophosphate esters (OPEs) are the derivates of phosphoric acid that comprise the same phosphate base unit (i.e., a central phosphate molecule and heterogeneous substituents) [1]. Based on their chemical structures, OPEs can be categorized into three groups: chlorinated OPEs, alkyl phosphates, and aryl phosphates [2]. OPEs are widely used in various commercial-, transportation-, and construction-based industrial products. Chlorinated OPEs are mainly used as flame retardants (FRs), and non-chlorinated OPEs are used as plasticizers [3]. Owing to the restrictions on the use of brominated flame retardants (BFRs), OPEs were used as the suitable substitutes since they are relatively environmentally friendly and have lower market prices [4]. It is reported that OPEs usage has increased rapidly in recent years [5,6]. The ubiquity of OPEs in the environment and their toxicities, such as neurotoxicity and reproductive genotoxicity, render them a potential threat to human health and the environment [7]. There are concerns regarding the impact of a large increase in the use of OPEs on the environment and human health since the environmental and health risks of OPEs are not fully understood yet [3]. Thus, elevated level of OPEs is an increasingly raised concern. Rather than chemically bonding to the materials, OPEs are physically mixed with polymer materials, causing their release into the environment via volatilization and leaching [8]. With the increasing use of OPEs as retardants and plasticizers, they are being detected worldwide in a wide range of environments, including water, sediment, air, outdoor and indoor dust, soil, and biota [9,10,11,12,13]. Many OPE-related studies were carried out in developing countries that either do not have strict regulations or do not enforce them [9]. Unlike BFRs, OPEs have not been listed as persistent organic pollutants by the Stockholm Convention [14]. Tri(2-chloroethyl) phosphate (TCEP) has gradually been replaced by tri(1-chloro-2propyl) phosphate (TCIPP) in some US and Europe regions, owing to the carcinogenic and mutagenic properties of TCEP [15].

With an increasing number of toxicological epidemiological studies, there has been increased concern about the adverse effects of OPEs on humans and other organisms. Being less persistent with low bioaccumulation and toxicity to humans and the environment than BFRs, as reported by the Scientific Committee on Health and Environmental Risks (https://ec.europa.eu/health/scientific_committees/environmental_risks_bg?2nd-language=it (accessed on 31 October 2021.) [16], OPEs have been used as an alternative for PBDE. The toxicities of OPEs have been gradually reported in recent years; OPEs are considered to have potentially carcinogenic, neurotoxic, genotoxic, mutagenic and reproductive effects [3,14,15]. The neurotoxic and carcinogenic effects of TCEP, TCIPP, and tri(1,3-dichloro-2-propyl) phosphate (TDCIPP) have been demonstrated, while the neurotoxicity of tri-phenyl phosphate (TPHP) is still under discussion [16]. TPHP and tris(2-butoxyethyl) phosphate (TBEP) have been reported to induce oxidative stress, DNA damage, and cardiotoxic [17]. In addition, 2-ethylhexydiphenyl phosphate (EHDPP)has been demonstrated to be cytotoxic and cause DNA damage and mitochondrial impairments [18]. The toxicity and ubiquity of OPEs in the environment and food indicate a potential threat to human health via the inhalation of air, dust ingestion, dermal contract, and dietary intake [19]. Herein, human health and environment threats from OPEs should remain of concern.

Recently, extensive scientific research regarding OPE has been published. However, there are limited reviews of OPEs. Current reviews concerning OPEs mainly focus on their environment occurrence, toxicity, and risk assessment [1,16,20]. Several studies reviewed the analytical methodology of OPEs, while only one study addressed the environmental occurrence of OPEs and other developing countries [1,21,22]. The occurrence of OPEs in indoor dust of China has been reviewed [14]. Considering these reports, we aim to provide a thorough, up-to-date review on the occurrence of OPEs in China. We have collected all possible available studies published over the last decade (from September 2009 to September 2021) from the Web of Science, ScienceDirect, Nature Springer, CNKI, and Google Scholar, using the following keywords: “organophosphate esters (OPEs)”, and “organophosphate flame retardants (OPFRs)”. We highlight the overall view regarding environmental levels of OPEs in different matrices as starting points to monitor their time trends in China. This review will discuss the production of OPEs to determine their potential release into the environment and the pathways of OPE release and provide valuable and detailed information regarding the occurrence and distribution of OPEs in various environments including water, sediment, soil, air, indoor and outdoor dust, and biota in different areas. The implications of ecological risk and human exposure are summarized in this review to make people realize and pay attention to the effects of OPEs. However, a wide environmental perspective outlines the analysis regarding the occurrence and distribution of OPEs. This information could provide an important reference for a better understanding of the occurrence and distribution of OPEs.

## 2. Methodology

This review aims to incorporate all the studies which have been published in the scientific literature regarding OPEs in various environments (water, sediment, dust, air, soil, and biota) in China. Hence, we searched for scientific works published from 2009 to 2021 using the following keywords “organophosphate esters”, “organophosphate flame retardants”, “OPEs”, “OPFRs”, in the most common online databases, namely Web of Science, ScienceDirect, Nature Springer, CNKI, and Google Scholar. There were 2399 search results in total. The following major inclusion criteria were used: (1) the studies must be published in a scientific journal; (2) those studies reported the fate, occurrence, ecological risk, and or human exposure or toxicity of OPEs in various environment (i.e., water, sediment, dust, air, soil, and biota); and (3) they must be referring to China (studies on organophosphate esters in other countries are not included). Using the selection criteria, we checked the search results manually by screening titles and abstracts, leaving 293 papers from web of science. After detailed data screening processes, we collected a total of 89 different available microenvironments (water n = 28, sediment n = 10, dust n = 21, air n = 11, soil n = 11, and biota n = 8) in China. The location, time, detailed matrix of sampling, the sum, and individual OPEs in different microenvironments are recorded. Concentration range, median and mean concentration, and detection ratio were collected.

The typical OPEs including three chlorinated OPEs (Tris(2-chloroethyl) phosphate (TCEP), Tris (2-chloroiso-propyl) phosphate (TCIPP), Tris (2-chloro, l-chloromethy-ethyl) phosphate (TDCIPP)), ten alkyl phosphates (Trimethyiphosphate (TMP), Triethyl phosphate (TEP), Tris(2,3-Dibromopropyl) phosphate (TDBPP), Tri(2-ethylhexyl) phosphate (TEHP), Tributyl phosphate (TNBP), Tri-tert-butyl phosphate (TiBP), Tributy phosphate (TBP), Tris(2-butoxyethyl) phosphate (TBEP), Tris(2-butoxyethyl) phosphate (TBOEP), and Tripropyl phosphate (TPP)), and four aryl phosphates (Tricresyl phosphate (TCrP), Tripheny phosphate (TPhP), 2-Ethylhexyl diphenyl phosphate (EHDPP), and Tricresyl phosphate (TMPP)) were summarized, since they were frequently encountered in the present studies.

The statistical analyses and data visualization were processed and analyzed with Excel 365 (Microsoft Inc., Remond, WA, USA), and Origin 9.0 (OriginLab Corporation, Northampton, MA, USA). The measurement units were unified to ng/L in water, ng/g in sediment and soil, and ng/m^3^ in dust and air. Water microenvironments were divided into rivers, lakes, marine water, wastewater, drinking water, snow, rainwater and road runoff. Dust and air microenvironments include houses, dormitories, libraries, offices, and special microenvironments such as instrumental houses and chemical laboratories. Soil samples primarily in farmland, industrial and residential areas, railways, and highways were available.

## 3. Fate of OPEs in China

The most common OPEs are presented in Table 1; their names, abbreviations, and properties are summarized. Most of the OPEs have a positive log Kow value, indicating that OPEs are more lipophilic than hydrophilic [23]. Some halogenated groups lead alkyl and aryl-ester groups different physico-chemical—ranging from very polar and volatile for trimethyl phosphate to non-polar and non-volatile for tri(ethylhexyl) phosphate [16,23]. Owing to the restriction on the use of brominated flame retardants (BFRs), as a substitute, OPEs is an increasing global market demand products. In 1992, the worldwide production and consumption of OPE was estimated as 102,000 tons, which increased to 210,000 tons in 2004. By 2006, the number of it doubled and increased to 465,000 tons [24,25]. The latest data suggests that the global annual consumption was up to 680,000 tons in 2015 and was forecasted to be 1,000,000 tons in 2018 [7,26]. China has more than 1850 FR manufacturers, accounting for 30% of the total global usages of OPEs. In China, the consumption of OPEs increased from 70,000 tons in 2007 to 100,000 tons in 2011, and it is estimated to increase at an average annual growth rate of 15% [7,14,27]. OPEs can be applied in many areas, such as in electric equipment, industrial materials, and building materials [28,29]. Chlorinated OPEs are used as flame retardants, and the nonhalogenated OPEs are used as plasticizers [30]. In addition, OPE can be used as additives in floor polish, lubricants, lacquers, hydraulic fluid, and as extractants for hydrometallurgy and nuclear energy [3].

Chlorinated OPEs are most widely used in rubber, flexible polyurethane foam (PUF), and FRs. Nonchlorinated OPEs, including TMPP and TPHP, are used in hydraulic and engine oils as plasticizers [1,31]. TNBP, TiBP, TBOEP, and TEHP are added into antifoam agents, hydraulic fluids, and coatings [27]. 2-Ethylhexyl diphenyl phosphate (EHDPP) is used in food packaging and paints (Table 2) [27].

OPEs are extensively used in various commercial products, which leads to their release from plasticizers, materials, vehicles, and electronic waste (e-waste) recycling activities into the indoor and outdoor environments [20,32]. Furthermore, there is a trend for the dispersion of OPE from the indoor environment to the outdoor environment via dust and air [1]. For instance, the OPEs in the indoor air can be accumulated in the clothes and then transferred to the outdoors environment via laundering and the normal wear loss cycles [33]. Abundant treated and untreated wastewaters affect surface water and groundwater through runoff and infiltration. The high effluent concentration of total OPEs was 2710 ng L^−1^ in the wastewater, and in the sludge, samples ranged from 14.2 to 136 ng g^−1^ in a wastewater treatment plant in the Pearl River Delta [34]. It was estimated that the total riverine input of OPEs was 16 ± 3.2 t yr^−1^ in the Bohai sea and the north Yellow Sea [35].

**Figure 1:**
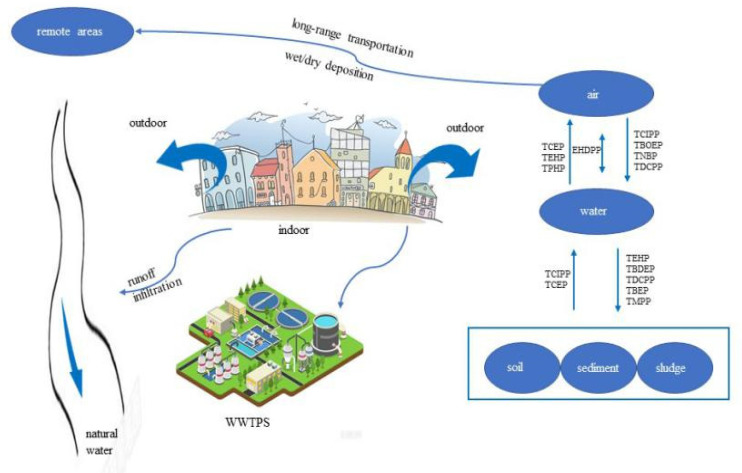
The pathway of common organophosphate esters (OPEs) in various environments.

The OPEs’ content in water and air reaches equilibrium through the air–water gaseous exchange and dry deposition. For example, the results obtained by calculating the net air–water exchange fluxes of OPE show that TCIPP and TBOEP were mostly deposited from the atmosphere to the water, in addition to TCEP, followed by TEHP, and TPHP primarily evaporated from water to the air [36]. TNBP and TDCIPP were principally deposited from the air into the water, while EHDPP reached an equilibrium between air and water via the fugacity fraction (ff) [31]. OPEs could also be originated from long-range transportation and then enter the water environments via dry/wet deposition. In northern China, the Hun River belongs to the Liao River system, and there are no obvious wastewater discharges from the domestics or industries. Therefore, the long-range transportation can be the only reason for OPE contamination in the Hun River [37]. OPEs can contaminate soil mainly due to the atmospheric deposition into the soil from the air. Simultaneously, the dispersion of gaseous OPEs can also make a slight difference (Figure 1) [38,39].

## 4. Occurrence of OPEs in China

Detailed information on the names, abbreviation, structures, and properties of OPE is listed in Table 1. Chlorinated OPEs include TCEP, TCIPP, TDCIPP, alkyl phosphates primarily include TMP, TEP, TDBPP, TEHP, TNBP, TiBP, TBP, TBEP, TBOEP, aryl phosphates include TPP, TCrP, TPhP, EHDPP, TMP. The analysis of data (Tables S1–S15, Supporting Information) revealed large concentration variations among microenvironments, including inflowing river water (range: 0.69–10.62 µgL^−1^), sediments (range: 0.0197–0.234 µg/g), dust (range: 8.706–34.872 µg/g), and open recycling sites’ soil (range: 0.122–2.1 µg/g). 

### 4.1. Water and Sediment

**Figure 2:**
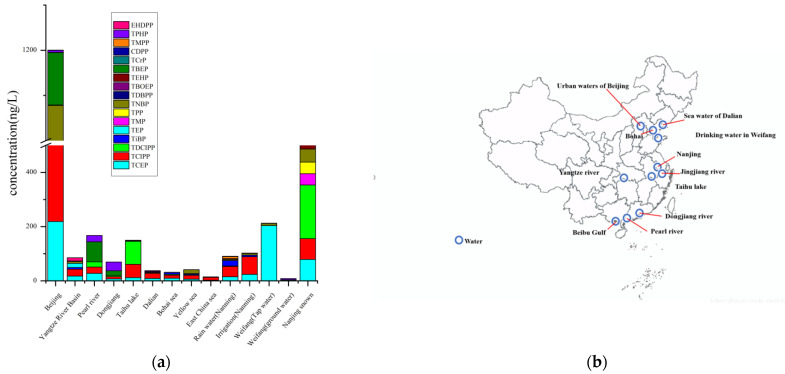
(**a**) Average concentrations (ng/L) of organophosphate esters (OPEs) in natural waters involving: rivers; lakes; marine water; groundwater; tap water. Mean values are used; detailed data are compiled in Tables S1–S3; (**b**) geographical distribution of organophosphate esters (OPEs) natural waters in China.

The concentrations of 17 selected OPEs in natural waters (rivers, seas, and lakes) are presented in Tables S1–S3. The total concentration of OPEs in aqueous environments are attributed to the different developing levels and industrial structures of different cities, since an important source of OPEs is wastewater discharge. For instance, samples from the Jinjiang River near the developed area of Chengdu showed the highest OPE levels ranging from 689.09 to 10,623.94 ngL^−1^, primarily polluted by Tris(2-butoxyethyl) phosphate (TBEP), human activities were pointed as the main source of OPEs pollution [40]. The maximum median concentration of ∑_12_OPEs was found as 1066 ngL^−1^ in the Fangting River (inflowing river) in Jiangsu province [41]. Relatively high OPE levels were found from the urban region of Beijing (Figure 2), in which the ∑_14_OPEs varied between 3.24 and 10,945 ngL^−1^. Tris (2-chloroiso-propyl) phosphate (TCIPP) and Tris(2-chloroethyl) phosphate (TCEP) showed high levels with average concentrations of 291 and 219 ngL^−1^, respectively [13]. High concentrations of alkyl phosphates such as TiBP and TNBP were found in the southern cities with higher economic levels [42,43]. Aryl phosphates for TBOEP and TMPP were seldom detected or detected at low concentrations.

Rivers can also be one of the major sources/pathways for pollutants entering the sea [35,44]. Further, the total concentrations of OPEs in seawater are related to the levels of development for different regions due to their high amounts of production and consumption [45]. For instance, the total concentration of OPE ranged from 9.6 to 1549 ngL^−1^, with an average of 300 ngL^−1^ in the rivers entering the Bohai Sea [35]. For several important marginal seas adjacent to the most developed and populated regions in eastern China, the total concentrations of seven OPEs ranged from 8.81 to 100 ngL^−1^, relatively high total concentrations of OPE were found in the Bohai Sea than the Yellow Sea and the East China Sea [35]. OPEs in the sea mostly originated from the ocean currents and riverine inputs [42]. For coastal water, the total concentrations of OPE ranged from 48.3 to 681 ngL^−1^. The coastal water showed higher total nitrogen and lower salinity than seawater primarily due to anthropogenic influence. This influence can cause OPEs entering into the sea via treated or untreated wastewater [42]. Areas adjacent to the sea were still not seriously polluted by OPEs. For instance, low ∑_7_OPEs levels were observed in natural water samples from the surrounding sea of China in the Bohai Sea, Yellow, and East China Seas. Water near the coastal areas is less polluted by OPEs; their dominant OPEs content are the same as other regions, and both chlorinated OPEs, such as TCEP, TCIPP, and TDCIPP, are the predominant OPE compounds [46]. In general, the total concentration of OPEs varied greatly in the river; notably, the alkyl- and aryl-OPEs were less than the halogenated-OPEs [43]. Here, its anthropogenic influence should be the main concern. 

The total concentration of OPE in urban surface water was generally higher than that in the surface water of rivers in the rural regions [47]. For instance, the total concentrations of OPE in the urban region of Beijing varied between 3.24 and 10,945 ngL^−1^ with a mean value of 954 ngL^−1^, which is significantly higher than the surface water in the rural region of Shanghai (range = 185–321 ngL^−1^; mean = 222 ngL^−1^) [13,48]. The total concentration of OPE in urban surface water (range = 339–1689 ngL^−1^; mean = 850 ngL^−1^) was approximately four times higher than that in the rural surface water [48]. Treated and untreated wastewaters, rainwaters, and road runoffs can also make urban surface water contain more OPEs [13]. The similarity of the composition profiles between the urban and rural surface waters indicated the emission of OPEs in the urban area may be the primary source of OPEs in the rural areas, such as the paddy fields [47]. Tris(2-chloroethyl) phosphate (TCEP) was the dominant compound in urban surface water, while in rural surface water, it was Tris (2-chloroiso-propyl) phosphate (TCIPP). These differences in the levels and patterns of OPEs may be attributed to the fact that the urban areas are considerably influenced by anthropogenic activities [49]. The wide use of both TCEP and TCIPP in various products should raise concern as they were more persistent in the environment [43].

OPEs can accumulate easily in bodies of water with low-velocity flow. The concentration of total OPEs ranged from 100 to 1700 ngL^−1^ in Taihu lake; both TEHP and TCEP were the predominant compounds and enriched in the sediment in Taihu lake and Beijing [50]. Lake waters in Beijing were more polluted by OPEs, with total concentrations of OPEs ranged from 3.24 to 10,945 ngL^−1^. OPE-related industries and wastewater treatment plants are key contributors to OPE pollution. Scarce data are available regarding OPEs in the Lake. Therefore, it is urgent to monitor the level of pollution in Taihu lake.

**Figure 3:**
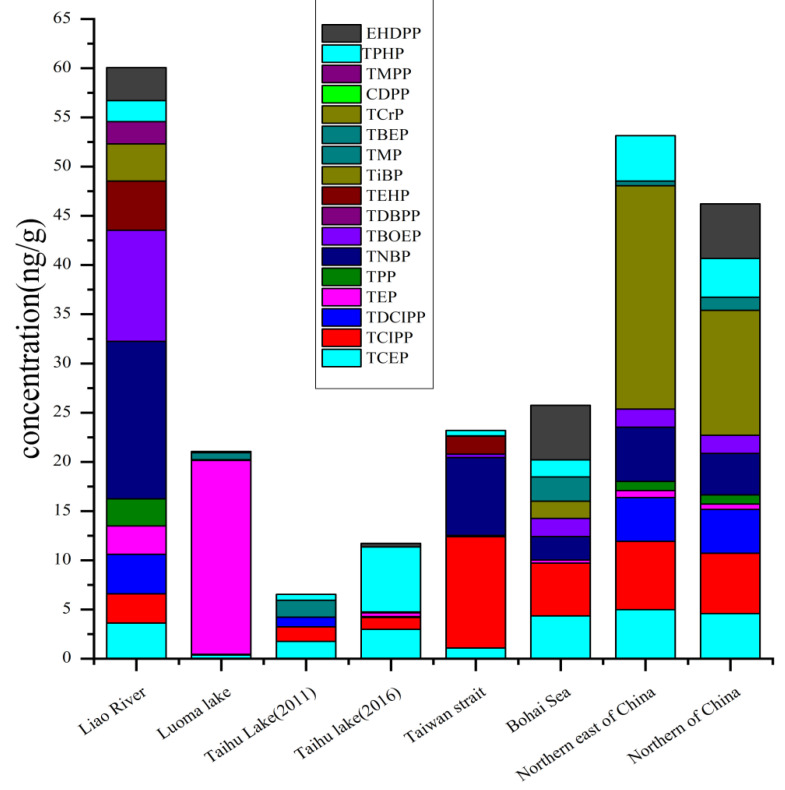
Average concentrations (ng/g) of organophosphate esters (OPEs) in sediments in China. The data of bar for Taihu Lake (2011) and Taihu Lake (2016) was available in 2011 and 2016. Mean values are used; detailed data are compiled in Tables S4–S6.

Sediment is an important sink of OPEs in the aquatic system [51]. For OPEs in the sediment, the concentration of total OPEs in surface sediments from the Liao River were relatively higher than the Taiwan Strait, the Bohai and Yellow Seas, and the Taihu lake [36,52,53]. For instance, the concentration of ∑_13_OPEs ranged from 19.7 to 234 ng g^−1^ in the Liao River in northeast China, with a mean concentration of 64.2 ng g^−1^ [27]. Liao River was primarily polluted by TiBP, TBOEP, and TNBP. The levels and spatial distribution of OPEs pollution in the Liao River was related to the domestic wastewater discharge, industrial activities, and agricultural production [52]. In 2011, a study measured the total level of OPEs (TCEP, TCIPP, TDCIPP, TPHP, TTP, TBEP, and TBP) in the sediments in Taihu Lake [52]. The results afforded by this investigation were different from those obtained in the study conducted in Taiwan [37]. This may be attributed to the different usage patterns of OPE in industry between mainland and Taiwan and the region close to the mainland of Taiwan Strait; TNBP, TEHP, and TCIPP were the predominant OPEs in the sediments. In the region adjacent to Taiwan Island, the chlorinated OPEs were dominant (Figure 3) [37].

Vertical distribution of OPE in the sediment cores can show the emission history of the OPEs [38]. In the coastal zone near the Yellow River Delta National Natural Reserve, the concentration of total OPEs in the surface water layer was high, especially for TNBP, TCEP, and TCIPP, indicating a significant increase in the use of OPEs [36]. Similar phenomena appeared in Taihu Lake; the level of total OPEs in 2016 was higher than that in 2011 in Taihu Lake, and the level of Tris (2-chloroiso-propyl) phosphate (TCIPP) increased [52,54]. Lake water can be used as drinking water. Thus, it is critical to monitor the levels of OPE in the lakes.

Sludge containing chemicals can act as a crucial indicator of industrial location and the usage of OPEs in local regions [55]. In 2014, the total concentration of seven OPEs ranged from 96.7 to 1312.9 ng kg^−1^ in sludge from the Pearl River Delta [55]. The total concentration of OPEs increased, ranging from 204 to 4101 ng kg^−1^ (dry weight, dw) (range = 128–2563 ng kg^−1^, wet weight, ww) in sludge from sewage treatment plants (STPs) in Beijing [56]. Relatively low total concentrations of OPEs were detected in the sludge from Henan province [57]. The level of OPEs was significantly associated with treatment capacity, processing volume, and the population served of sewage treatment plants [57]. Sludge samples from municipal and industrial STPs contain high levels of nonchlorinated OPEs [58]. For instance, the sludge from STPs in Beijing was contaminated by Tri(2-ethylhexyl) phosphate) (TEHP) and Tricresyl phosphate (TCrP), with an average concentration of 233 and 137 ng/g, respectively, which was attributed to their strong absorption tendency and the use pattern of different OPE [56].

OPEs enter wastewater treatment plants via domestic sewage, industrial wastewater, and dry and wet deposition from the surface water and atmosphere [59]. The total concentrations of OPEs ranged from 65.8 to 2842 ngL^−1^ in influent samples and 6.37 to 2710 ngL^−1^ in effluent samples from industrial wastewater treatment plants [35]. In a municipal wastewater plant in the Pearl River Delta, the concentrations of TBP and TBEP were up to 21,271.8 and 4349.4 ngL^−1^ in influent wastewater and 3105.1 and 494.5 ngL^−1^ in the effluent wastewater, which primarily originated from OPEs-containing products [29]. The removal efficiencies for the nonchlorinated OPEs were high in the municipal wastewater treatment plant (WWTP) in Pearl River Delta. For instance, the removal efficiencies of Tributy phosphate (TBP), Tris(2-butoxyethyl) phosphate (TBEP), and Tripheny phosphate (TPhP) were up to 85.6%, 88.7%, and 87.8% [29]. However, still quite high concentrations of nonchlorinated OPEs in the range of 25.6–3108.8 ngL^−1^ remained in the effluent and the chlorinated OPEs were not removed. On the contrary, their concentration increased slightly [29]. Hence, the output of OPEs from the wastewater treatment plants should raise concern. At the wastewater treatment plant, OPEs were removed in the anaerobic zone more effectively than the sedimentation and secondary sedimentation tanks and pre-anoxic, anoxic, and oxic zones [59]. Therefore, the upgrading and reconstructing WWTP to reduce the levels of OPEs may be a trend in the future.

Drinking water treatment processes cannot remove OPEs completely [25]. In the cities, the concentration of total OPEs in tap water were 3.99–192 ngL^−1^ [60]. For some dominant OPEs in the tap water, the mean concentrations of TBEP, TPP, and TCIPP were 70.1, 40.0, and 33.4 ngL^−1^ [61]. The reported level of total OPEs in drinking water are about one order of magnitude lower than those in natural water (Figure 2). Compared with natural water and wastewater, different concentrations of OPEs and abundant Triethyl phosphate (TEP) were reported in the tap water, while similar distribution levels of OPEs, primarily TCIPP, TDCIPP, and TBEP were reported in the well water and barreled water [60,61]. Tap water showed much higher concentration levels than bottled water and well water [61]. However, human exposure to OPEs through drinking water is not a serious threat [60]. Although the levels of OPEs in the drinking water are still low, the issue is proposed to be of concern.

In snow samples only from the urban and suburban areas of Nanjing, the total concentration of 11 OPEs ranged from 229.1 to 1175.0 ngL^−1^ [62]. The OPEs primarily comprised TDCIPP (35.3–460.0 ngL^−1^), TBEP (3.0–579.1 ngL^−1^), TCEP (58.9–126.0 ngL^−1^), TCIPP (25.6–173.0 ngL^−1^), and TEHP (12.1–219.0 ngL^−1^). OPEs in the snow mainly come from long-distance migration and atmospheric dry and wet deposition [62]. Rainwater and road runoff can reflect the level of OPEs in the dust and air [13,63]. Considerably high concentration of Tris(2-chloroethyl) phosphate (TCEP) in the rainwater ranged from 2270–3560 ngL^−1^ in Beijing, and Tris (2-chloroiso-propyl) phosphate (TCIPP) in the road runoff was detected at 846–1578 ngL^−1^, which indicated that road dust in Beijing was seriously polluted by OPE [13]. Hence, rainfall, which acts as an important carrier of OPE contamination in the environment, should also be concerning.

### 4.2. Dust and Air

#### 4.2.1. Indoor and Outdoor Dust

**Figure 4:**
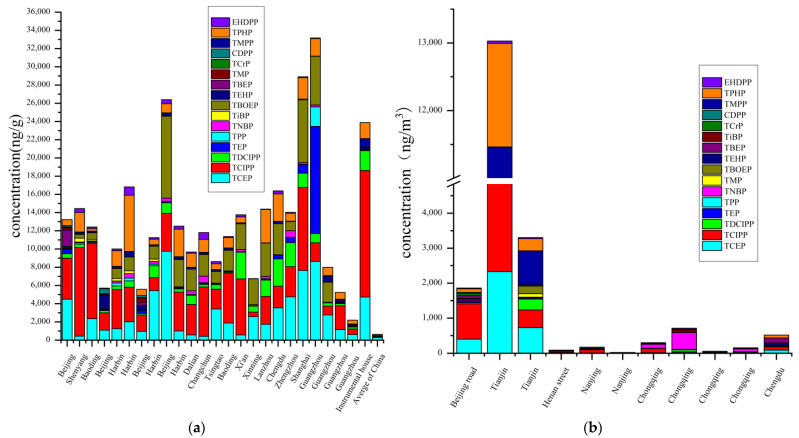
(**a**) Concentrations of organophosphate esters (OPEs) in indoor dust from different microenvironments including office, library, dormitory, residential home, chemical laboratory, instrumental house. Mean values are used; detailed data are compiled in Tables S10–S12; (**b**) concentrations (ng/m^3^) of organophosphate esters (OPEs) in outdoor dust from different microenvironments. Mean values are used; Detailed data are compiled in Tables S10–S12.

Indoor environments are major sources for human exposure to OPEs; people typically spend >20 h per day in indoor environments [64]. Owing to the large-scale production and subsequent use of OPEs in consumer products, high levels of OPEs in the indoor dust were reported [14]. In this context, microenvironments play an important role in the distribution of OPEs. The levels of each OPE in indoor dust from several microenvironments including houses, dormitories, libraries, offices, and special microenvironments such as instrumental houses and chemical laboratories are compiled in Figure 4 and Tables S10–S12. 

The occurrence of OPEs in the indoor dust is related to the type and amount of furniture, building materials, electronic appliances and the general degree of ventilation [21]. Dust from public building microenvironments (e.g., schools, hotels, hospitals, restaurants, and theaters) contains much higher levels of OPEs than domestic buildings microenvironments due to stricter fire safety regulations, requiring FRs, and more usage of products containing OPEs [65]. The total concentration of OPE in indoor dust ranged from 0.292 to 9.540 μg g^−1^ in northwestern and southwestern China [66]. The total concentrations (mean value: μg g^−1^) of OPE in dust were 14, 5.9, and 6.9 in office, residential homes, and dormitories in Beijing [67]. A new study found that the levels of OPEs in the newly decorated apartments were significantly higher than dormitories and computer rooms in Dalian [68]. Dust samples from dormitories showed low OPE, which is likely explained by the simple furniture, decoration in dormitories, and frequent ventilation [64]. The total concentrations (median value = 14.354 μg g^−1^) of 10 OPEs were in a range of 8.706–34.872 μg g^−1^ in libraries, in which the dominant OPE were TCEP, TBOEP, and TCIPP. These values may be attributed to the type of ornamentation, furniture, electronic appliances, and flooring used in the library [68]. Regarding special microenvironments, high levels of OPEs were obtained in houses comprising analytical instruments and having less ventilation [69]. The highest concentrations of OPEs ranged from 3.30–70.0 μg g^−1^ with a median of 25.0 μg g^−1^ in the e-waste recycling workshops in Qingyuan, which is about three times than in the dormitories. This is related to a long history of using OPEs as FRs and additives [70]. High level of OPE in the e-waste recycling sites’ indoor dust and higher estimated daily intake of OPE from this indoor dust than outdoor dust could be a serious health threat for local workers and residents. However, outdoor waste recycling shows a lower risk for workers [54].

The high level of Tris(2-butoxyethyl) phosphate (TBOEP) in the indoor dust was consistent with the regular use of floor polishes and waxes or PVC and rubber products in residential houses [69]. TCEP and Tris (2-chloroiso-propyl) phosphate (TCIPP) were mainly used in rigid and flexible polyurethane foam (PUF) applicants, such as upholstery and furniture [71]. High TPhP levels in e-waste sites indicate the use of TPhP in electrical products, but the relationship between the e-waste types and pollution extent is not clear [72]. High concentration (median value = 9.57 μg g^−1^) of Tris (2-chloroiso-propyl) phosphate (TCIPP) was attributed to the use of products containing OPEs, such as wire and cable insulation, connectors, automotive interiors, and hydraulic fluids [70]. Tris (2-chloro, l-chloromethy-ethyl) phosphate (TDCIPP) is used in furniture only in a small fraction; thus, TDCIPP was found at low concentrations [73]. Hence, it is important to continue monitoring the exposure of OPEs via various indoor dust microenvironments.

The levels of OPEs in outdoor environments are relatively lower than indoor dust owing to fewer sources [28]. Relatively high concentrations of OPEs were found in the urban roads in Beijing (0.933–11.293 μg g^−1^) than the industrial roads in a suburban area of Chongqing (0.0488–0.476 μg g^−1^); traffic and population may be the major sources for the higher OPE concentrations [74]. It has verified the hypothesis that industrial emissions were responsible for the OPEs in road dust [8,28,75]. Dry deposition due to the high vapor pressure of alkyl OPEs (TMP, TEP, and TnBP) played an important role in the distribution of OPEs in outdoor dust [76]. The lower ∑OPEs concentrations are due to new development and smaller scale of digital electronic commerce in downtown Chengdu downtown resulting in lower OPE-related risk [74]. The highest concentration of total OPEs in outdoor dust detected was in the multi-waste recycling area in Jinghai district, Tianjin (Figure 4). Chlorinated OPEs (TCIPP, TCEP, and TDCIPP), aryl-OPEs (TPHP and TMPP), and TBOEP were found at the highest concentrations originating from abandoned automobiles, e-waste, electric wire, plastics, and rubber products [24,54]. Studies outdoor dust containing OPEs have been sparse and should be considered as an important exposure source, warranting additional studies.

#### 4.2.2. Air

Air is regarded as the main reservoir for the OPE released from various products, which results in the existence of OPEs in the atmosphere [20]. OPEs were found predominantly in the particle phase since OPEs show low vapor pressure and high affinity for particles. It also pointed out that there is a limited partition of OPEs to the gas phase since rare OPEs were found in some gas samples [2]. Hence, studies focused more on the particle-bound concentrations (PM2.5, total suspended particulates) of OPEs in the air [2,3,77,78]. The levels and distribution patterns of the OPEs are attributed to the types and quantities of emission sources, including households, vehicles, light industries [2,78].

**Figure 5:**
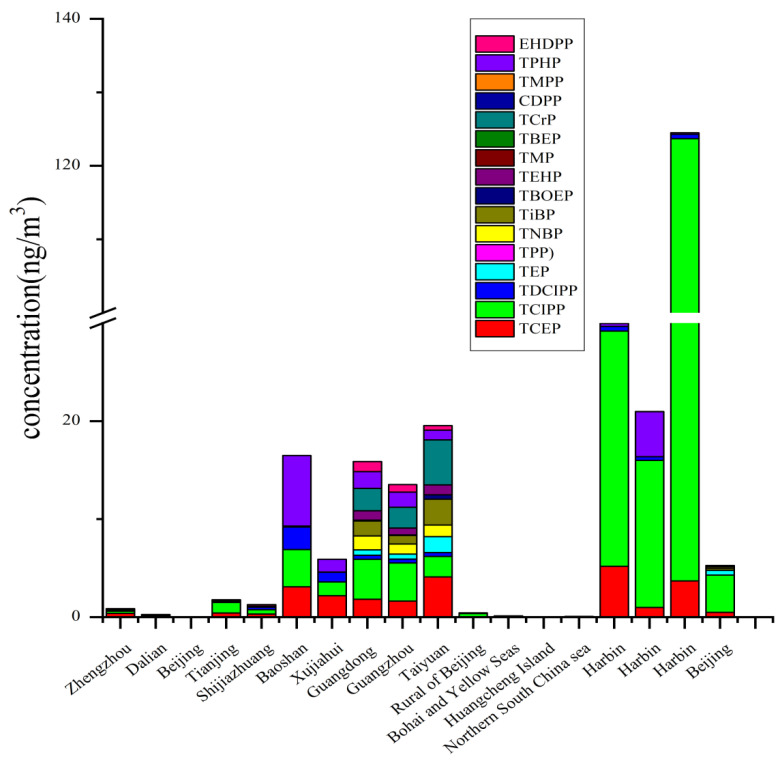
Concentrations (ng/m^3^) of organophosphate esters (OPEs) in air from different microenvironments. Mean values are used; detailed data were displayed (Tables S7–S9).

As presented in Figure 5, high concentrations of OPEs were obtained in the urban regions of Guangzhou and Taiyuan. The total concentrations of OPEs ranged from 3.10 to 544 ng/m^3^, and chlorinated OPEs were the dominant OPEs, which may be due to more volatilization during the production and application of OPEs [14]. Higher concentrations of OPEs were found in a city park (3050 pg m^−3^) than rural site (0.79 pg m^−3^) in Dalian due to the population or heavy traffic [1]. High levels of OPEs in were observed in urban and suburban sites compared to observations in rural sites in Tianjin were observed, due to the population density and products containing OPEs [79]. The coastal area and remote sites tended to have lower concentrations of total OPEs compared with other urban environments, such as residential areas, universities, commercial and traveling areas, high-tech development zones, and industrial and traffic centers [31,78,79,80]. For instance, the lower concentration of ∑OPEs was 0.36–0.21 ng m^−3^ in Dalian, where air–soil diffusion plays an important role in controlling the levels of OPEs in the air [31]. Wind direction can influence the accumulation of OPEs in the air. For some special sites, e-waste dismantling plants, phosphorus-containing FR manufacturing plants, and airport which were identified as significant sources of OPEs and are crucial points of concern.

The studies on indoor air are rare, probably due to the fact that the levels of OPE in the gas samples were fewer in some previous reports [31,67]. The indoor environments were more severely polluted by OPEs. The total concentration of OPEs in the indoor air was 1–2 orders of magnitude higher than the outdoor air. For instance, the concentrations of ∑_12_OPEs (median = 9.1 ng/m^3^) in a test home (including three bedrooms, one living room, one kitchen, and two bathrooms) in Harbin were ~6–9 times higher than Beijing–Tianjin–Hebei region outdoor air samples [31,81]. The concentration of Tris (2-chloroiso-propyl) phosphate (TCIPP) in the living room in Harbin (mean = 120 ng/m^3^) was >100 times higher than the outdoor air in Zhengzhou (mean = 0.22 ng/m^3^), Dalian (mean = 1.2 ng/m^3^), Tianjin (mean = 1.08 ng/m^3^) [31,39,76,81]. Indoor air concentrations (mean) of OPEs in decorated apartment microenvironments in Dalian showed higher mean concentrations (23.2 ng/m^3^), followed by 6.62, 5.59, 3.01, and 2.90 in a computer room, office, garage, and dormitory [68]. Considerably higher levels of OPEs in the indoor air than the outdoor air may be attributed to the common usage of OPE in furniture, building materials, textiles, and plastics [67]. Low total concentration of OPE (1.0–20 ng m^−3^, mean = 5.2 ng m^−3^) was detected in the indoor air in dormitories and offices in Beijing due to the use of simple furniture [67].

The concentrations (mean, median, and range) of OPEs in the air from different areas are compiled in Figure 5 and Tables S7–S9. In the outdoor air, seasonal variations of the emission potential and the environmental occurrence of OPEs during hot seasons influence the level of OPEs. The alkylated and chlorinated OPEs were dominant both in summer and winter [79]. Generally, relatively higher levels of gaseous-phase OPEs were found in summers than in winters, TDCIPP with low detectability was not included, while higher levels of Tripheny phosphate (TPhP) and Tri(2-ethylhexyl) phosphate (TEHP) were found in the particle-bound phase in winter [31,82]. Season variations differ between indoor air and outdoor air. Indoor air due to low total suspended particle (TSP) concentrations showed no elevated OPE concentrations in summer. OPE concentrations in the indoor air were different from the indoor dust in office, the concentrations of OPEs in the indoor dust were higher in winter than in summers. On the one hand, this difference may be attributed to the fact that lower temperature makes OPEs remain on the dust particles instead of volatilizing into the air. On the other hand, more/less frequent ventilation accelerates/slows the OPEs migration from dust into the air in different seasons [67,81]. For some northern areas, such as Dalian, one of the reasons for higher levels of OPEs in the winters than in summers may be the presence of central heating in winter [68]. Inhalation is expected to be an important intake pathway for human exposure to OPE. Hence, the OPE contamination of the air should be concerning.

### 4.3. Soil

**Figure 6:**
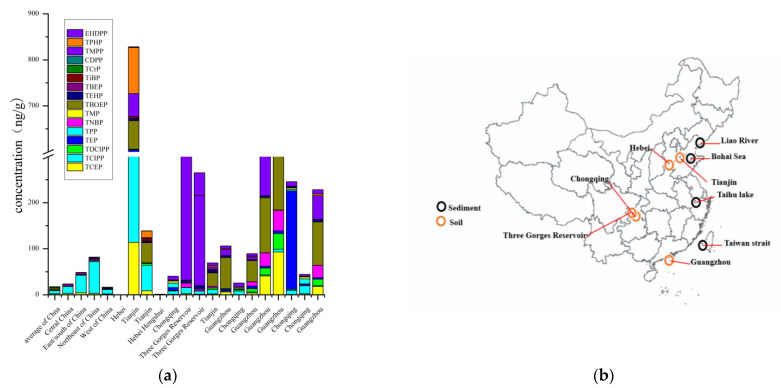
(**a**) Mean concentrations (ng/g) of organophosphate esters (OPEs) in soil from China (mean values are used); detailed data is presented in Supplementary Data Tables S10–S12; (**b**) geographical distribution of organophosphate esters (OPEs) sediment and soil in China.

Soil is a likely sink for various organic pollutants, including OPEs. Limited studies are available on the levels and distributions of OPEs in soil matrices. The latest data indicated that the highest concentration of ∑_12_OPEs was observed in farmlands around open recycling sites of Tianjin with the range of 122–2100 ng g^−1^ (mean = 829 ng g^−1^), which mainly related to the release of OPEs from household materials (Figure 6) [54]. Relatively high concentrations of OPEs were detected in commercial areas (green land soils of the road greenbelts) with heavy traffic and extensive anthropogenic activities in the roadside soil samples of Guangzhou (range = 410–1370 ng g^−1^; mean = 250 ng g^−1^) [10]. The total concentrations of OPEs in the farmland soils were high owing to the agricultural activities, such as wastewater irrigation and organic matter [26]. Tris(2-butoxyethyl) phosphate (TBEP) exhibited the highest concentration among all the OPEs in Hebei, Guangzhou, and Chongqing [83,84]. Tripheny phosphate (TPhP) is the dominant OPE in the e-waste dismantling areas since it is added into materials physically and thus prone to be released from the e-waste products [85,86]. In general, soils were investigated and are primarily polluted by chlorinated OPEs. Chlorinated OPEs can easily accumulate in soil due to their low solubility, high absorption potential, and persistent behavior [75]. For nonchlorinated OPEs, TBOEP, TMPP, TNBP, TPHP, TEHP, and TCEP are abundant in Guangzhou, due to the heavy traffic and extensive anthropogenic activities in textile, building, and electrical commercial areas [10,54,87]. 

Low levels of OPEs were detected in Tibetan Plateau, the median concentrations of OPEs were 69.1 ng/g, 17.5 ng/g, 14.7 ng/g, and 11.1 ng/g in Lhasa (farmland soil), Nagri (apaleochannel in a desert soil), Namtso (meadow near a lake soil), and Nyingchi (coniferous forest soil) [88]. The soil in Chongqing (industrial area, commercial area, residential area, and city park) showed less contamination with OPEs; the total concentration of OPE was in the range of 10.7–108 ng/g, with a mean concentration of 55.6 and 55.5 ng/g in the industrial and residential areas, and 41.8 and 34.1 in the commercial area and city park, respectively. There were multiple sources of OPE contamination in urban soils of Chongqing [89]. It may be attributed that the sample soil is away from the sources, for instance, the soil, in cities, including factories, railways, highways, showed low levels of OPEs, which are about one to two orders of magnitude lower than high levels [75]. Residential areas were less polluted than urban areas or areas closed to point sources [75]. For some special soils, riparian areas of the Three Gorges Reservoir Region (upper reach of Three Gorges Reservoir Region) were polluted more seriously than farmland, this may be attributed to the anthropogenic activities with the full operation of the Three Gorges Reservoir Region [90]. The occurrence and distribution of OPEs in the soil may influence the biotic and abiotic soil functions, the quality of crops, and the health of animals and humans. Hence, the contamination of OPEs in the soil should be concerning.

### 4.4. Biota

**Figure 7:**
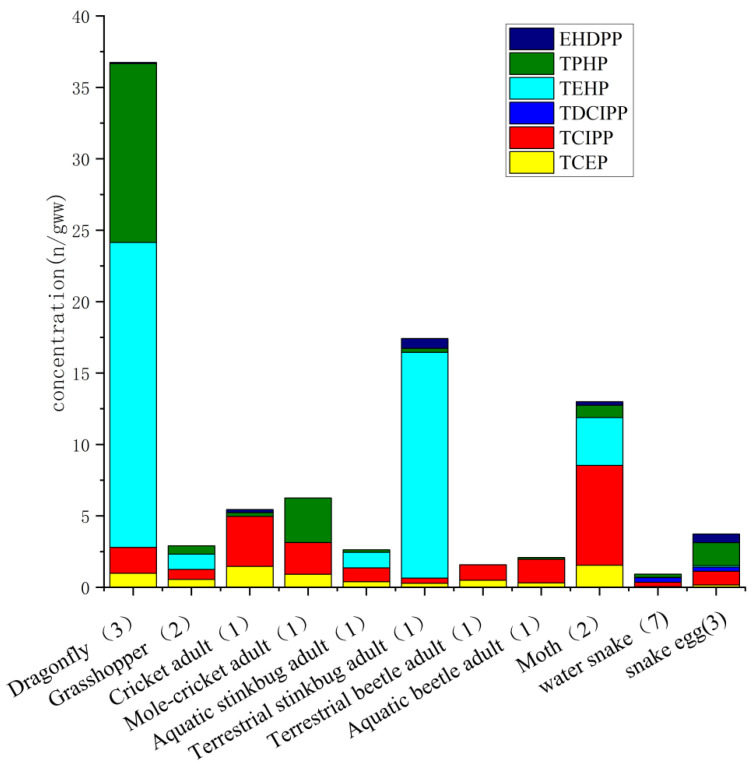
Average concentrations of organophosphate esters (OPEs) in biota samples, including snake (South China); fish (Pearl River delta, South China, Laizhou Bay); and insects (a pond and farmland of Longtang in Guangdong). Mean values are used; detailed data are compiled in Tables S16–S18.

The migration of OPEs in the environments makes it exposed to biota [91]. However, it is difficult to separate OPEs from the complex biological matrix [92]. Studies on the occurrence of OPEs in biota are scarce in China. To our knowledge, limited information is available on the occurrence of OPEs in biota samples including snakes, aquatic organisms, and insects (Figure 7, Tables S16–S18). The level of total OPEs in the biota ranged from 1.8 to 22 ng/g wet weight (ww) [92]. Different biotas exhibited different patterns of OPEs contamination. For vertebrates, the mean concentrations (ng/g ww) of ∑OPE were detected at 1.9 and 12 in water snake muscles and snake eggs in the dismantling site of south China. They were dominated by TCIPP, TNBP, and TPHP [93]. For aquatic organisms, such as topmouth gudgeon, crucian carp, loach, and catfish have been investigated in Beijing and Pearl River delta, and fish from Pearl River delta contained extremely high OPEs, particularly for TBEP (range: 164–8842 ng/g), TCEP (range: 82.7–4692 ng/g), and TNBP (43.9–2946 ng/g), which was about 100 times higher than marine fish species from Laizhou Bay [92,94]. Except for aquatic organisms, only one study reported the levels of OPEs in the insects, the most contaminated analyzed insects were dragonfly nymphs, followed by moth adults (25.7 ng/g ww) and terrestrial stinkbugs (17.4 ng/g ww) [95]. The insects were less influenced by OPEs compared with aquatic organisms. For instance, near the e-waste recycling site in Guangdong, the total concentration of OPEs was 67.8 ng/g ww, which was lower than aquatic organisms numerically [95]. Analyzed insects were mainly polluted by Tri(2-ethylhexyl) phosphate) (TEHP), with an average concentration of 5.8 ng/g ww, followed by TPHP (2.5 ng/g ww), TCIPP (2.2 ng/g ww), TCEP (0.8 ng/g ww), and EHDPP (0.1 ng/g ww) [95]. These levels of OPEs were lower than snakes and aquatic organisms (relatively speaking). More studies reported trophic biodilution rather than biomagnification of OPEs. For example, a study that collected water snake and small common carp samples from the same Chinese pond found that the biomagnification factor was 0.13, suggesting that there was a biodilution rather than biomagnification of OPEs in the water snake. Additional studies reported the levels of OPEs in muscle tissues of biota, TEHP, TBP, TBOEP, and TPHP were the dominant OPEs in the fish tissues [92]. Except comparing different biotas, the maternal transfer in the same biota was complicated. The concentrations of OPEs and the change in chemical composition between water snake eggs and muscle were different. For instance, TCEP, TCIPP, TDCIPP, and TEP showed higher proportions in the muscle, while TNBP in the water snake eggs is higher [93]. On the one hand, the mechanism of maternal transfer of OPEs in reptilian species is complicated. On the other hand, a lack of data on the levels and trends of OPEs in the biotas makes it difficult to compare the concentrations among the same or different biotas. Hence, we need more studies to research. 

Limited information is available on the occurrence of OPEs in human samples including human whole blood [96], plasma [97], placenta [98], serum [96], and urine [96,99,100,101,102]. Whether organophosphate diester (di-OPE) can reflect human exposure to all OPEs with great differences in chemical structures and properties is under discussion. Biomakers are used to assess human exposure to OPEs, since they can reflect integrated exposure including different sources and pathways [96]. Eight OPEs and detailed the ranges, and the median concentrations of the organophosphate esters (OPEs) in human are available and listed in Table 3. In the investigated adults in Beijing, the level of OPEs in the whole blood (range/median concentration: 2.61–79.2/8.63 ng/mL) and serum (range/median concentration: 1.08–20.4/5.71 ng/mL) is higher than in the urine (range/median concentration: 0.106–11.6/0.396 ng/mL) [96]. Tri(2-chloroethyl) phosphate (TCEP) was dominant in human placentas, with a median concentration of 142 ng/g lw (lipid weight), followed by tributoxyethyl phosphate (TBEP) and triphenyl phosphate (TPhP) [98]. The values of total concentration of 12 OPEs ranged from ND (not detected) to 8.84 µg/L and ND to 20.11 µg/L in hypertensive patients’ and unpaid blood donors’ plasma, with average concentrations of 0.62 µg/L and 1.46 µg/L [97]. In the human body, OPEs can be hydrolyzed and excreted into organophosphate diester (di-OPE) in urine as dialkyl or diaryl phosphote metabolites rapidly [101]. The concentration of total OPEs in urine (adults in Beijing) was in the range of 0.106–11.6 ng/mL, with median concentration of 0.396 ng/mL [96]. Urinary biomarkers of OPEs (tri (1,3-dichloro-2-propyl) phosphate (TDCPP), tris (1-chloro-2-propyl) phosphate (TCIPP), tri-n-butyl phosphate (TNBP), and tris (2-chloroethyl) phosphate (TCEP)), were also frequently detected with median concentrations of 6.2, 1.5, 2.6 and 0.3 ng/mL in adolescent students’ urine in Hangzhou [99]. These results demonstrated that OPEs and their metabolites are available in human; however, information on the levels of OPEs in human is limited. Comprehensive investigations on the bioaccumulation and biomagnification of OPEs in the human are required to clarify their risk assessment. 

## 5. Risk Assessment

To assess ecological risk, OPE concentrations in various environments have been evaluated in numerous studies. The risk quotient (*RQ*) method was used to assess the ecological risk of a chemical on aquatic organisms and ecotoxicological risks of residual OPEs in water, sediment, and soils [7,27,103]. The *RQ* was calculated using the following equation:

(1) $${RQ}_{i}=\frac{{MEC}_{i}}{{PNEC}_{i}}=\frac{{MEC}_{i}}{L(E)C_{50}/f}$$

where *MEC* is the measured environmental concentration of OPEs in surface water, sediments, and soil; *PNEC* refers to the predicted no-effect concentration of OPEs; *L*(*E*)*C*_50_ is the lowest median effective concentration value obtained from the reports; and *f* is a security factor, which is equal to 1000 [7,15,103]. The *L*(*E*)*C*_50_ and *PNEC* values were obtained from previous studies [7,13,14]. The common risk ranking types (criteria) were low risk (0.01 < *RQ* < 0.1), moderate risk (0.1 < *RQ* < 1), and high risk (*RQ* > 1) [50]. In this study, the ecological risks from nine typical OPEs, namely TCEP, TCPP, TCIPP, TMP, TCrP, TNBP, TiBP, EHDPP, and TPHP, were assessed for aquatic organisms (crucian carp) and soil (Table 4) due to their high levels.

The *RQ* values were calculated based on Equation (1) and the measured concentration values of OPE in biotas (Tables S16–S18) and soils (Tables S13–S15). Considering the discrepancies in soil microenvironments, we selected the report investigating nationwide soils. The individual *RQ* values for the nine selected OPEs are presented in Table 4. The individual OPE *RQ* values are in the range of 0–2.60 × 10^−^^1^ in aquatic organisms and 0–8.53 × 10^−3^ in soils. In general, most single OPEs showed low risk (<0.1) for aquatic organisms in water, except for a moderate risk (0.26) posed by Tributylphosphate (TNBP) to crucian carp. In Dongting, the risks of TCEP and TCIPP for algae, crustaceans, and fish were much lower than 0.1, indicating low ecological risk. The *RQ*s of Tripheny phosphate (TPhP) ranged between 0 and 0.056, suggesting a limited but higher ecological risk than others [6]. In addition, the risk quotients of EHDPP, TNBP, and TDCPP should be concerning, since their mean *RQ*s values exceeded 0.1 [27]. For aquatic organisms, the variety of organic pollutants in the water and sediments affecting the ecological risk need to be paid much closer attention. In addition, the available *PNEC* values of OPEs for other biotas remain unknown, which will cause an underestimation of the associated risk.

For soil, the *RQ*s of chlorinated OPEs showed the same order of magnitude between 0 and 10^−^^1^ (Table 4). Nonhalogenated OPEs showed lower *RQ*s than chlorinated OPEs; the lowest *RQ* of close to 10^−6^ indicates limited ecological risks to the soil. However, for detailed areas, such as Tianjin, the highest *RQ* values were found from multiwaste recycling areas: 0.05–17.2 in Tianjin; some industrial areas showed moderate risk (4.35 × 10^−2^) [7]. Tricresyl phosphate (TMPP) and Tripheny phosphate (TPhP) with *RQ* > 1 showed high risk in multiwaste recycling areas in Tianjin and Jinan, implying their high toxicity [7,54]. There is still inadequate information available to assess the soil ecological risks. Hence, more investigations regarding OPEs in the soils are necessary. The ecological risks vary from different *PNEC*s and relevant environmental concentrations. The *PNEC*s for TNBP, TiBP, TBOEP, and TEHP were not available, leading to some uncertainty in the soil ecological risks [104].

OPEs can be transported from wastewater to surface water in aquatic environments [14]. When aquatic organisms are exposed to environmentally relevant concentrations of OPEs, OPEs such as tricresyl phosphate (TCrP), triphenyl phosphate (TPhP), and 2-ethylhexyldiphenylphosphate (EHDPP) maybe concerned can be bioaccumulated and biotransformed in aquatic organisms [94]. Some high OPEs concentrations in the water and sediment suggest that these OPEs may cause various toxic effects on aquatic organisms, including reproductive and development toxicity, genotoxicity, neurotoxicity, acute, organ toxicity, mutagenicity, and endocrine disruption. It was demonstrated that OPEs can be accumulated in animals and insect [95]. Insects, as a dominant component of biodiversity, play a key role in the initial steps of bioaccumulation of OPEs from the environment [95]. High concentrations of OPEs can be accumulated in gulls through the diet and further transfer them to eggs [105]. A large amount of OPEs can be introduced into farmland soil when irrigating with reclamed wastewater and application of biosolids. Plants, such as wheat, could take up OPEs and accumulate them from contaminated soil, water and sediment by roots and translocate them to branches, leaves, and fruits [106]. The pathway for human exposure via wheat would cause a threat to human health [106]. Children exposed to OPEs more seriously due to frequent hand-to-mouth activities, lower body weight, lower breathing zone, and more time spend indoors [107]. Due to vulnerable immature metabolic, neurologic, and immune systems of children, OPEs exposure in early life stage should be a significant health concern.

## 6. Human Exposure

Ubiquitous OPEs exist in various environments, which attributes human exposure to OPEs mostly through three pathways consisting of absorption, oral intake of food and dust, and inhalation [64]. Owing to investigating only a single microenvironment or the discrepancies in the same microenvironments in most studies to evaluate exposure, the exposure estimates and comparisons become complicated and uncertain [19]. Thus, we summarized common health risk assessment of air, dust, and water and discussed the relatively higher and lower levels of risk in China. OPEs in indoor dust and air could be indicators to assess human exposure and health risk. Estimated daily intake (EDI) of OPEs is used to assess human exposure to OPEs on drinking water and indoor dust and air exposure. The calculation of *EDI* in different environments showed a little difference.

*EDI*s of OPEs in air inhalation, dust ingestion, and drinking water.

(2) $${EDI}_{air\;inhalation}=\left(\frac{C_{air}\times V_{air}}{BW}\right)$$

(3) $${EDI}_{dust\;ingestion}=\left(\frac{C_{dust}\times DIR}{BW}\right)$$

(4) $${EDI}_{drinking\;water}=\left(\frac{C_{water}\times IR\times AP}{BW}\right)$$

where *C* is the concentration of OPEs measured in air (ng/m^−3^), indoor dust (ng g^−1^), or water (ng L^−1^), *V_air_* is the volume of air, *DIR* is the dust ingestion rate (g day^−1^), *BW* is the body weight (kg), and *IR* and *AP* are ingestion rates of water (L day^−1^) and absorption percent of drinking water, respectively [38,67,108]. 

### 6.1. Casual Environmental Exposure

OPEs can be accumulated in the soil and dust. Human exposed to OPEs in various environment mostly occur by ingestion, inhalation and dermal. People can ingest OPEs via soil or dust when putting their hands and other objects into their mouths. Children are more seriously exposed to OPEs due to more frequent hand-to-mouth contact [2]. The EDI for both the children and adults exposure to OPEs via air inhalation of air was at least three orders of magnitude lower than the reference dose values [67]. High EDI values of 35 and 6.7 ng kg^−1^ day^−1^ for airborne exposure for toddlers and adults were found in Beijing [67]. Low levels of OPEs (0.07–1.14 and 0.03–0.50 ng kg^−1^ day^−1^) for toddlers and adults were detected in Taiyuan and Guangzhou [109]. Although human exposure to OPEs only posed low health risks, chronic toxicities are still unknown. It should be noted that children spending more time in indoor environments showed 2–11 times higher exposure levels of EDIs than adults [67]. For instance, the EDI of OPEs in adults was estimated to be 1.6 ng kg^−1^ day^−1^, and 31.7 ng kg^−1^ day^−1^ for children in South China [110]. For air inhalation, the inhalation of OPEs ranged from 5.85 to 350 pg kg^−1^ day^−1^ in the urban cities (Beijing, Tianjin, and Shijiazhuang) [8]. Inhalation of OPE-contaminated air with EDIs levels for human exposure did not pose a significant health risk for humans. 

To assess health risks, human exposure to OPEs in the dust environment via dermal absorption has been estimated in many studies. The choices of microenvironments, sampling strategies, and dust particle size, varied from study to study, thus, affecting exposure estimates. Therefore, the comparisons of exposure assessment results are complicated [19]. Human exposure to OPEs via dust, the highest EDIs of OPEs (median levels) were estimated at 0.42–14.3 and 6.09–208 ng kg^−1^ day^−1^ for adults and children in e-waste recycling sites in villages of South China [72]. Therefore, there is a local public health threat from OPEs nearby e-waste regions. Relatively low EDI values of total OPEs (median levels) were found in environments different than the e-waste regions, including urban street dust in Henan (0.0101 ng kg^−1^ day^−1^ for adults and 0.0603 ng kg^−1^ day^−1^) for children and campus road dust in Chongqing (0.26 ng kg^−1^ day^−1^ for adults and 0.53 ng kg^−1^ day^−1^) for children [111,112]. Although the EDIs of OPEs for both adults and children were generally 2–4 orders of magnitude lower than the corresponding reference doses, it should remain of concern that the average dust ingestion of children was nearly 20 times higher than adults [110].

The EDIs of individual OPE were at least two orders of magnitude lower than the corresponding reference doses [41,61]. The relatively high EDIs of ∑_11_OPEs for adults and children in Nanjing via drinking water ranged from 26.6 to 39.0 ng kg^−1^ day^−1^) [62]. The investigated EDIs of OPEs in different types (tap water, filtered drinking water, well water, bottled water, barreled water) of drinking water ranged from 0.14 to 7.07 ng kg^−1^ day^−1^. It is still not a critical issue for human exposure to OPEs through drinking water, but drinking water ingestion is one of the important ways for human exposure to OPEs. Although most of the EDIs of OPEs indicated a limited human health risk, it was notable that under the worst scenario, the highest concentration of TCEP and TBOEP showed a possible risk to children [69]. Food ingestion, face mask, hand wipes, and other exposure pathways should also be concerned to determine the human health risk in further studies [113,114].

### 6.2. Occupational Exposure

Human exposed to OPEs in office dust and e-waste workshops is more serious than that in other microenvironments [2]. For workers, especially in the e-waste recycling sites, abundant e-waste is dismantled in workshops or even in living rooms. Indoor dust ingestion, poor hygiene awareness multiply OPEs exposure risk [72]. Hence, dust ingestion and hand-to-mouth contact were the primary exposure pathways [115]. For electronic engineers, undergraduates, and graduate students, the EDI via floor dust ingestion were 1.37, 0.75 and 1.24 ng kg^−1^ day^−1^ under the mean exposure scenario in Nanjing, respectively [15]. Tripheny phosphate (TPhP) showed the highest EDI for dust ingestion (6.88 ng kg bw^−^^1^ day^−^^1^) in a waste dismantling area [115]. For students in dormitories, college dormitories have smaller personal space normally and comprised of less furniture and electronic appliances, the median EDIs (ng/kg-day) of total OPEs were 2.45 and 2.15 for female and male students, respectively [64]. The occurrence of OPEs on workers’ hand can be as a relevant occupational exposure assessment, potential risk or decreased exposure regarding the usage of mask for workers haven’t been studied. 

## 7. Perspectives

The contamination levels of OPEs in different environments in China have been summarized in this study for future monitoring of trends over time. The EDIs of OPEs by air inhalation, dust ingestion, and drinking water were compiled. Based on the present review, four major research gaps have been presently identified and summarized.

First, although an increasing number of studies have recently reported the distribution and occurrence of OPEs and their production and consumption in developed areas, the studies on the use and emission of OPEs are lacking. The occurrence and environmental fate of OPEs in different environments (water, sediment, soil, air, dust, and biota) have been investigated and the concentration of OPEs varied among different years, even within the same region. However, the differences between the methods, years, and investigation of individual OPEs were not considered in this study. A criteria inventory of OPEs reflecting the levels of OPEs contamination is needed in China for better investigation.

Second, OPEs exist in various environments, including water, sediments, soil, sludge, dust, and air. However, previous studies have focused on one type of environment or the interaction between two (water and sediment, soil and dust) environmental media. The occurrence of OPEs in the indoor dust and air were studied synthetically more, and data between the indoor microenvironments and biotas, such as fish and plants, were sparse. Future studies should focus on the levels of OPEs in the houseplants or ornamental fish indoor to better understand the risk of human exposure to OPEs. 

Third, nowadays, the available studies focused on the legacy OPE contaminants (TCEP, TDCIPP, TCIPP, TBEP, TEHP, TNBP, TPHP, etc.) in various environments and risk assessments. Evidence from these legacy OPEs has raised public awareness of risks regarding human health. The emerging OPEs are expected to increase in the future. However, there remains a paucity of information on the occurrence, distribution, and risk assessment of the emerging OPEs. We need to conduct more research on the emerging contaminants, focusing on one or two classes of chemicals.

Finally, the extensive use of OPEs makes them exist in various environmental media, which the human body is closely related to, including food, water, dust, air. Studies on human exposure to OPEs should be a long time and a sound risk assessment would request regular monitoring of programmers in the environment, food, matrices supported by human biomonitoring studies, and epidemiological evidence. Not only direct exposure to OPEs through air inhalation, dust ingestion, and dietary intake is concerned, such as food and food processing both make human exposure to OPEs more, but the biotransformation should also be taken into consideration seriously. We can work on the human metabolites to access the risk of human exposure to OPEs in a long time. Still, the EDI of human exposure to OPEs is lower than the reference dose values. More studies should be conducted to discover the potential impact of OPEs on human health, particularly for children.

## 8. Conclusions

Over the past decade, an increasing number of studies have focused on OPEs in the environment, owing to their wide use and toxicity. In summary, this review primarily has provided an overall view about environmental levels of OPEs in different matrices as a starting point to monitor time trends. OPEs are ubiquitous in water and indoor dust, especially in areas adjacent to pollution sources. The concentration of OPEs in water is higher in highly developed cities than in rural areas. Dust from e-waste sites and public buildings is more seriously polluted by OPEs. With the increasing use of OPEs, the emissions of OPEs from consumer products will also increase. Moreover, with more strict regulations of flammability standards and the urbanization of more cities, the need for OPEs will increase in many developing areas. OPEs have been physically incorporated into many products, which causes them to release into the environment (air, dust, water, and soil) through volatilization, diffusion, and abrasion. 

Humans can be exposed to OPEs primarily through ingestion of contaminated food or dust, inhalation of contaminated air, and dermal contact with air, dust, or soil. People spend a significant time indoors (e.g., homes, offices, library, and workshops), and dust from public building (e.g., schools, hotels, hospitals, restaurants, and theaters) contains much higher levels of OPEs than domestic buildings due to stricter fire safety regulations, requiring flame retardants, and more usage of products containing OPEs. This can affect human health adversely as a result of acute, reproductive, and developmental toxicity, neurotoxicity, organ toxicity, genotoxicity, mutagenicity, and endocrine disruption. Other exposure pathways such as face masks, hand wipes, or phone contact may also be a cause for concern. The EDI for human exposure to OPEs is lower than the reference dose values, but the EDI for children was higher than that for adults due to their lesser exposure time indoors and their lesser body weight. Children are exposed to OPEs more serious via dust ingestion and inhalation than adults, which should be concerning.

## Figures and Tables

**Table 1:** The names, abbreviation and properties of the most common in the present study.

Name	Abbreviation	Structural Formula of OPEs	P	log Kow	S
Tris(2-chloroethyl) phosphate	TCEP	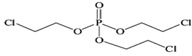	1.1 × 10^−4^	1.44	7.00 × 10^3^
Tris (2-chloroiso-propyl) phosphate	TCIPP	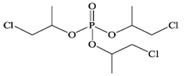	1.9 × 10^−6^	2.59	1200
Tris (2-chloro, l-chloromethy-ethyl) phosphate	TDCIPP	^ 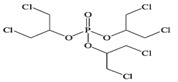 ^	7.4 × 10^−8^	3.65	7
Trimethyiphosphate	TMP	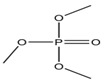	-	0.65	-
Triethyl phosphate	TEP	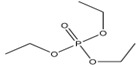	0.29	0.80	5.0 × 10^5^
Tris(2,3-Dibromopropyl) phosphate	TDBPP	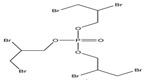	-	4.05	-
Tri(2-ethylhexyl) phosphate)	TEHP	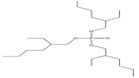	2.0 × 10^−6^	9.49	0.6
Tributyl phosphate	TNBP	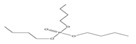	1.1 × 10^−3^	4.00	280
Tri-tert-butyl phosphate	TiBP	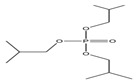	1.3 × 10^−2^	3.6	160
Tributy phosphate	TBP	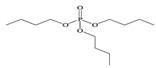	-	4.0	280
Tris(2-butoxyethyl) phosphate	TBEP	^ 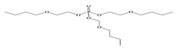 ^	-	3.75	1100
Tris(2-butoxyethyl) phosphate	TBOEP	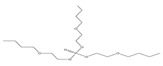	2.1 × 10^−7^	3.65	1.20 × 10^3^
Tripropyl phosphate	TPP	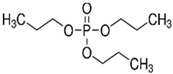	2.9 × 10^−2^	2.12	827
Tricresyl phosphate	TCrP	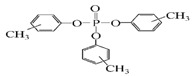	-	5.11	-
Tripheny phosphate	TPhP	^ 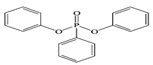 ^	1.2 × 10^−6^	4.59	1.9
2-Ethylhexyl diphenyl phosphate	EHDPP	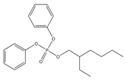	6.5 × 10^−7^	6.64	1.9
Tricresyl phosphate	TMPP	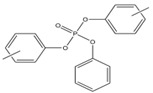	0.03	5.11	0.36

P: vapor pressure (mmHg) at 25 °C; S: water solubility (mg/L) in water 25 °C; log Kow: n-octanol/water partition coefficient.

**Table 2:** The application of organophosphate esters (OPEs).

Abbreviation	Application
TCEP	Flame retardants, plasticizers, flexible, rigid polyurethane, soft foams, wallpapers, paints
TCIPP	Flame retardants, plasticizers, flexible, rigid polyurethane, insulation, sealant foams
TDCIPP	Flame retardants, plasticizers, flexible, rigid polyurethane
TNBP	Foam, textiles, polystyrene panel, hydraulic fluids
TiBP	Antifoaming agent
TBEP	Floor wax and polishes, plastics, rubber products
TEHP	Flame retardants, plasticizers
TMPP	Plasticizers, hydraulic fluids, engine oils
EHDPP	Food packaging, paints
TPHP	Plasticizers, hydraulic fluids, PVC, lubricants, electronic equipment, building materials
TCrP	Cutting fluid
TBOEP	Floor polishes, nonwoven wallpaper, rubber products, cutting oil
TPP	Polyurethane foam

**Table 3:** Concentrations (mean/median, µg/L) of the individual and total concentration (range, µg/L) organophosphate esters (OPEs) in whole blood, plasma, placenta, serum, and urine.

Region	Cohort	Sample Year	TEP	TCIPP	TPhP	TMPP	TEHP	EHDPP	TNBP	TCEP	ΣOPE	Reference
Hubei	Hypertensive patients (plasma n = 232)	2019	2.38/1.10	0.10/ND	0.01/ND	0.55/0.79	0.01/ND	0.66/0.71	ND/ND	-	ND-8.84	[97]
Hubei	Healthy unpaid blood donors (plasma n = 56)	2019	1.84/0.16	0.28/ND	6.49/6.01	0.01/ND	0.28/ND	0.56/ND	0.76/0.88	-	ND-20.11	[97]
Beijing	Adults (whole blood samples n = 57)	2018	0.430/0.432	NC/<0.166	0.352/0.366	NC/<0.026	NC/<0.145	0.862/1.100	0.184/0.176	NC/<0.194	2.61–79.2	[96]
Beijing	Adults (serum samples n = 57)	2018	0.175/0.196	0.648/1.05	NC/<0.307	NC/<0.026	NC/<0.145	0.714/0.933	NC/<0.145	NC/<0.194	1.08–20.4	[96]
Beijing	Adults (urine samples n = 52)	2018	0.087/0.075	NC/<0.033	NC/<0.061	NC/<0.002	NC/<0.091	NC/<0.016	NC/<0.091	NC/<0.039	0.106–11.6	[96]
Eastern China	Mothers (placenta n = 25) ^a^	2005	10.2	ND	15.1	-	ND	ND	ND	142	34.4–862	[98]

ND: not detected. NC: not calculated due to the low detection frequencies (<50%). ^a^: only median concentrations (ng/g liquid weight) were available. -: data were not available.

**Table 4:** Ecological risk data of typical organophosphate esters (OPEs) in the aquatic species, soil, and dust in China.

Compound	Microenvironments	MEC (ng/g)	L(E)C_50_ (mg/g)	PNEC (ng/g)	RQs in China	References
TCEP	*Carassius auratus auratus* ^a^	105.72	90	90,000	0–1.17 × 10^−3^	[91]
	soil ^c^	3.07	0.386	386	0–7.95 × 10^−3^	[91]
TCPP	*Carassius auratus auratus* ^a^	117.75	30	30,000	0–3.92 × 10^−3^	[91]
	soil ^c^	14.5	1.700	1700	0–8.53 × 10^−3^	[104]
TCIPP	*Carassius auratus auratus* ^a^	13.57	5.1	5100	0–2.66 × 10^−3^	[91]
	soil ^c^	1.36	0.320	320	0–4.25 × 10^−3^	[104]
TMP	pimephales promelas ^d^	144.2	7000	7000	0–2.06 × 10^−5^	[1]
	soil ^c^	nd	nd			
TCrP	*Carassius auratus auratus* ^a^	nd	0.11	110	0–1.17 × 10^−3^	[101]
	soil ^c^	nd	nd			
TnBP	*Carassius auratus auratus* ^a^	228.78	8.8	880	0–2.60 × 10^−1^	[101]
	soil ^c^	2.56	nd			
TiBP	*Carassius auratus auratus* ^b^	103	20	20	0–5.15 × 10^−3^	[14]
	soil ^c^	0.28	nd			
EHDPP	crustacean ^d^	1.35	0.018	18	0.075	[27]
	soil ^c^	0.18	30.2	302	0–5.96 × 10^−4^	[104]
TPhP	*Carassius auratus auratus* ^a^	56.55	0.7	700	0–8.07 × 10^−2^	[91]
	soil ^c^	0.21	130	130,000	0–1.61 × 10^−6^	[104]

^a^ the measured environmental concentration (MEC) is the measured organophosphate esters (OPEs) concentrations in aquatic organisms (ng/g); the lowest median effective concentration value (L(E)C_50_) data are obtained from [13]. ^b^ the measured environmental concentration (MEC) is the measured organophosphate esters (OPEs) concentrations in aquatic organisms (ng/g), the lowest median effective concentration value (L(E)C_50_) data are obtained from [14]; ^c^ the measured environmental concentration (MEC) is the measured organophosphate esters (OPEs) concentrations (median concentration) in soil of nationwide in China (ng/g) [104], the lowest median effective concentration value (L(E)C_50_) data are obtained from [7]; ^d^ the measured environmental concentration (MEC) and the lowest median effective concentration value (L(E)C_50_) data are obtained from the relevant references of Table 4.

## Data Availability

Not applicable.

## Supplementary Materials

The following are available online at https://www.mdpi.com/article/10.3390/toxics9110310/s1. Table S1: Comparison of concentrations (range; arithmetic mean/median; Detection ratio% ng/L) of the total organophosphate esters (OPEs) and the predominant chloroalkyl phosphates in water from various water environments in China; Table S2: Comparison of concentrations (range; arithmetic mean/median; Detection ratio% ng/L) of the total organophosphate esters and the predominant Alkyl phosphate in water from various water environments in China; Table S3: Comparison of concentrations (range; arithmetic mean/median; ng/L) of the total organophosphate esters and the predominant Aryl phosphate in water from various water environments in China; Table S4: Comparison of concentrations (range; arithmetic mean/median; ng/g) of the total organophosphate esters (OPEs) and the predominant chloroalkyl phosphates in sediment and sludge from various sediment environments in China; Table S5: Comparison of concentrations (range; arithmetic mean/median; ng/g) of the total organophosphate esters (OPEs) and the predominant Alkyl phosphate in sediment from various sediment environments in China; Table S6: Comparison of concentrations (range; arithmetic mean/median; ng/m3) of the total organophosphate esters (OPEs) and the predominant Aryl phosphate in sediment from various sediment environments in China; Table S7: Comparison of concentrations (range; arithmetic mean/median; Detection ratio% ng/m3) of the total organophosphate esters and the dominant chloroalkyl phosphates in air from various air environments in China; Table S8: Comparison of concentrations (range; arithmetic mean/median; Detection ratio% ng/m3) of the total organophosphate esters and the predominant Alkyl phosphates in air from various air environments in China; Table S9: Comparison of concentrations (range; arithmetic mean/median; Detection ratio% ng/m^3^) of the total organophosphate esters (OPEs) and the predominant Aryl phosphate in air from various air environments in China; Table S10: Comparison of concentrations (range; arithmetic mean/median; detection ratio ng/g) of the total organophosphate esters (∑OPEs) and the predominant chlorinated phosphates in dust from various dust environments in China; Table S11: Comparison of concentrations (range; arithmetic mean/median; ng/g) of the total organophosphate esters (OPEs) and the predominant Alkyl phosphates in dust from various dust environments in China; Table S12: Comparison of concentrations (range; arithmetic mean/median; ng/g) of the total organophosphate esters and the predominant Aryl phosphate in dust from various dust environments in China; Table S13: Comparison of concentrations (range; arithmetic mean/median; ng/g) of the total organophosphate esters and the predominant chloroalkyl phosphates in soil from various soil environments in China; Table S14: Comparison of concentrations (range; arithmetic mean/median; ng/g) of the total organophosphate esters (OPEs) and the predominant Alkyl phosphate in soil from various soil environments in China; Table S15: Comparison of concentrations (range; arithmetic mean/median; ng/g) of the total organophosphate esters and the predominant Aryl phosphate in soil from various soil environments in China; Table S16: Comparison of concentrations (range; arithmetic mean/median; ng/g ww) of the total organophosphate esters and the predominant chloroalkyl phosphates in biota in China; Table S17: Comparison of concentrations (range; arithmetic mean/median; ng/g ww) of the total organophosphate esters (OPEs) and the predominant Alkyl phosphates in biota in China; Table S18: Comparison of concentrations (range, arithmetic mean ng/g ww) of the total organophosphate esters (OPEs) and the predominant Aryl phosphates in biota in China.
