# Supplementary material for: Organophosphate Esters in China: Fate, Occurrence, and Human Exposure
Source: Toxics. 2021 Nov 16;9(11):310. doi: 10.3390/toxics9110310 (PMC8620853; doi:10.3390/toxics9110310)
Supplement: Supplementary file 1 [file toxics-09-00310-s001.zip › toxics-1451310-supplementary.pdf]

toxics MDPI

Supplementary Materials: Organophosphate Esters in China: Fate, Occurrence, and Human Exposure

Zhihui Hu, Lingshi Yin, Xiaofeng Wen, Changbo Jiang, Yuannan Long, Jiawei Zhang and Ruyi Liu

Table S1. Comparison of concentrations (range; arithmetic mean/median; Detection ratio% ng/L) of the total organophosphate esters (OPEs) and the predominant chloroalkyl phosphates in water from various water environments in China.

Location	Description of matrix (sample year)	∑OPEs (the total number)	TCEP	TCIPP	TDCPP	Reference
			River water			
Shanghai (28)	Urban area (Jun 2018)	339-1689;<br>850/-<br>(8)	67.5-865.2;<br>348.5/340.1;<br>100%	123.9-523;<br>259/250.3;<br>100%	<11.53-45.3;<br>25.1/24.8;<br>96.4%	[1]
Shanghai (28)	Rural area; Chongming Island (Jun 2018)	185-321;<br>222/-<br>(8)	30-63.3;<br>38.2/36.4<br>100%	60-154.2;<br>77.4/70;<br>100%	<11.53;<br><11.53/<11.53;<br>0%	
Beijing (340)	Urban surface water including the river and lake water (2013-2014)	3.24-10945;<br>954/569;<br>(14)	<LOD-5698;<br>219/104;<br>95.8%	<LOD-1742;<br>291/174;<br>99.4%	<LOD-3617;<br>116/22.8;<br>90.2%	[2]
Chengdu	Jinjiang	689.09-10623.94;<br>3747.58/-<br>(7)	27.68-273.10<br>;-/-;<br>100%	35.76-143.75;<br>-/-;<br>100%	nd	[3]
Guangzhou	Pearl river	55-577;<br>235.86/183.5;<br>(7)	3.54-75.4;<br>27.78/19.2;<br>-	2.47-62.6;<br>23.37/15.25;<br>-	1.82-73.6;<br>19.13/6.94;<br>-	[4]
	Dongjiang river	24-293;<br>136.04/110.5;<br>(7)	1.47-16.2;<br>7.37/6.81;<br>-	3.96-12.9;<br>8.43/8.75;<br>-	1.54-6.68;<br>3.81/3.97;<br>-	
Yangtze River Basin	Mainstream	66.5-112;<br>83.4/79.7;<br>100% (13)	<LOD-22.5;<br>17.8/17.6;<br>93.8%	12.6-44.6;<br>25.2/25.3;<br>100%	<LOD;<br><LOD/<LOD;<br>0	[5]
	Inflowing rivers	55.6-5071;<br>357/176;<br>100% (13)	11.0-1202;<br>79.9/39.1;<br>100%	14.0-450;<br>91.5/67.7;<br>100%	<LOD-288;<br>39.5/11.5;<br>15.9%	
Yi River (1)	Inflowing river (Nov 2016)	97.1<br>(12)	-;<br>36.1/-;<br>-	-;<br>2.79/-<br>-	-;<br>0.40/-;<br>-	[5]
Fangting river (1)	Inflowing river (Nov 2016)	1066<br>(12)	-;<br>794/-;<br>-	-;<br>3.59/-;<br>-	-;<br>0.78/-;<br>-	
			Lake water			
Luoma Lake (14)	Include the estuary and	0.82-708;<br>127/73.2;<br>(12)	0.01-552;<br>69.9/24.3;<br>100%	0.02-10.8;<br>6.29/5.79;<br>100%	0.03-1.98;<br>0.95/1.03;<br>100%	[6]

	lake outlet (Nov 2016)					
Taihu Lake （25）	Taihu Lake and its tributaries(Nov 2016)	$1.0×10^2$-$1.7×10^3$； $8.0×10^2$/-； （12）	14-76； 44/47； 100%	12-$2.9×10^2$； 93/63； 100%	Nd-6.0; 1.8/1.4; 92%	[7]
Taihu Lake (29)	(Jun 2016)	166-1530; -/- (11)	31.6-1870; 1.12/-; 96%	59.7-12300; 449/-; 88%	9.74-682; 0.385/-; 92%	[8]
Marine water						
Dalian (14)	Bohai Sea and Yellow Sea urban district (Sep 2017)	21.6-61.5; 39.7/-; (9)	2.2-17.5; 8.74/7.93; -	11-26.3; 19.15/21.2; -	0.61-6.91; 2.63/1.82; -	[9]
Beibu Gulf	Culture ponds;	47.1-227; 122/116; (11)	-	-	-	[10]
	Estuaries; nearshore areas	32.9-71.3; 51.1/50.0; (11)	-	-	-	
Bohai Sea	Surface and bottom layer	20.85-56.59; 36.04/36.39; (7)	6.67-16.19; 9.81/10.17; -	4.76-17.4; 11.36/11.46; -	1.58-4.78; 3.02/2.87; -	[11]
Yellow Sea		12.47-73.91; 33.99/29.51; (7)	1.72-15.14; 6.30/5.75; -	6.2-30.8; 14.13/13.04; -	0.74-6.75; 2.63/2.47; -	
East China Sea		10.63-38.71; 17.00/15.11 (7)	0.78-8.07; 2.59/2.24; -	6.38-22.46; 10.67/9.16; -	0.16-4.06; 1.45/1.48; -	
Nanning	Rain water	-; 84±55/- (11)	-; 15/-; 100%	-; 38/-; 100%	-; 2.1/-; 83%	[12]
	Irrigation	-; 110±90/- (11)	-; 24/-; 100%	-; 65/-; 100%	-; 0.007/-; 83%	
Drink water						
Weifang (8)	Tap water	162-253; 215/218; (6)	153-238; 204/212; 100%			[13]
Weifang (4)	Ground water	3.52-13.9; 8.83/8.97;(6)	1.55-6.64; 3.4/2.58; 100%			
Sewage treatment plant (STP) influent waste water						
Pearl River Delta	Industrial STP1()	346	79.9	112	10.5	[14]
	STP2	2842	86.2	128	26.4	
	STP3	610	113	185	LOQ	
	STP4	361	LOQ	70.0	LOQ	
	STP5	305	33.1	62.5	95.7	
	STP6	65.8	19.2	14.0	LOQ	
	STP7	1065	126	638	LOQ	

	STP8	412	23.7	102	8.91	
Shanghai[a]	STP1(Jun 2018)	2142.546	218.22	289.61	30.20	[1]
	STP2(Jun 2018)	881.77	186.45	314.57	44.15	
	STP3(Jun 2018)	634.04	106.05	225.73	111.79	
	STP4(Jun 2018)	1325.82	329.044	555.66	77.60	
	STP5(Jun 2018)	1111.02	120.11	306.19	417.38	
	STP6(Jun 2018)	1196.43	521.75	426.67	57.86	
	STP7(Jun 2018)	2406.40	227.75	545.88	136.64	
Sewage treatment plant (STP) effluent waste water						
Pearl River Delta	Industrial STP1()	245(9)	17.1	58.5	9.09	[14]
	STP2	2710(9)	31.9	87.3	21.5	
	STP3	135(9)	17.2	56.5	7.90	
	STP4	50.9(9)	LOQ	34.1	1.74	
	STP5	69.9(9)	13.1	26.4	10.0	
	STP6	37.2(9)	13.5	14.1	LOQ	
	STP7	6.37(9)	LOQ	5.40	LOQ	
	STP8	202(9)	31.5	104	8.92	
Hebei Heng Shui	Manufacturing plant (Dec 2016)	7100-33000; 20000/20000 (4)	3000-15000; 8900/8900	4100-18000; 11000/11000		[15]
Shanghai[a]	STP1(Jun 2018)	752.53	215.65	295.43	47.63	
	STP2(Jun 2018)	818.99	205.45	267.39	36.48	
	STP3(Jun 2018)	469.26	77.97	186.42	45.55	
	STP4(Jun 2018)	852.95	222.12	366.35	55.73	
	STP5(Jun 2018)	610.10	133.36	277.31	36.05	
	STP6(Jun 2018)	1057.19	606.68	278.21	49.52	
	STP7(Jun 2018)	810.60	170.50	318.39	40.14	
Snow						
Nanjing	Urban area (Jan 2019)	229.1-1175.0; 746/858; (11)	58.9-126.0; 79.1/72.5; 100%	25.6-173.0; 76.8/59.0; 100%	35.3-460.0; 198/157; 100%	[16]

The full names for OPEs are summarized in Table 1. -:data not available. nd: not detected.

Table S2. Comparison of concentrations (range; arithmetic mean/median; Detection ratio% ng/L) of the total organophosphate esters and the predominant Alkyl phosphate in water from various water environments in China.

Location	TiBP	TEP	TMP	TPP	TNBP	TDBPP	TBOEP	TEHP	TBEP	Reference

River water										
Shanghai (28) urban	10.8-68.8; 27.7/20.6; 100%				11.6-63.3; 29.9/23.3; 100%				15.9-100.6; 46.6/46.2; 100%	[1]
Shanghai (28) rural	9.29-45.1; 29.8/30.1; 100%				6.91-44.8; 25/24.7; 100%				<16.6-47.9; 23.6/20.3; 67%	
Beijing (340)	<LOD-169; 23.3/14.1; 93.6%	<LOD-2072; 88.7/47.5;94.7%	<LOD-9497; 144/48.3;88.2%		<LOD-256; 19.6/10.1;84.9%			<LOD-23.5; 0.91/<LOD; 41.7%	<LOD-3617; 116/22.8; 90.2%	[2]
Chengdu					36.1685.41; -/-; 100%			30.55-143.75; -/-; 100%	274.25-10186.61; -/-; 100%	[3]
Pearl river									<LOD-200; 73.98/62.35; -	[4]
Dongjiang river									3.38-45.1; 17.46/14.73; -	
Yangtze river Basin	3.90-9.58; 6.51/3.68; 100%	10.4-30.9; 15.1/13.8;100%	<LOD; <LOD/<LOD; 0	<LOD; <LOD/<LOD; 0	4.8-8.86; 6.98/6.90; 100%			<LOD-2.10; 1.91/1.90; 87.5%;	<LOD; <LOD/<LOD;0	[5]
	1.50-3032; 55.7/7.91; 100%	<LOD-602; 30.1/13.6;96.6%	<LOD-48.2; 47.8/47.8; 2.3%	<LOD; <LOD/<LOD; 0	2.43-1132; 34.3/10.4; 100%			<LOD-23.9; 3.77/2.16; 96.6%	<LOD-3156; 116/3.75; 38.6%	
Yi River		-; 10.1/-; -	-; 43.1/-; -		-; 0.76/-; -	-; 0.64/-; -		-; 0.44/-; -	-; 0.03/-; -	
Fangting River		-; 5.66/-; -	-; 253/-; -		- 1.81/-; -	- 0.68/-; -		-; 0.43/-; -	-; 0.05/-; -	[6]
Lake										
Luoma Lake		<9-32.1; 11.7/10.5; 88%	<10-127; 25.2/16.4; 88%		0.01-5.85; 2.37/1.81; 100%	<0.70; 0.38/0.65; 63%		0.05-1.73; 0.79/0.95; 100%	0.02-10.8; 5.79/6.29; 100%	
Taihu Lake（25）		53-1.4×10^3; $6.2\times10^2/\times10^2$ 100%	2.7-84; 28/23; 100%		0；0/0；0%	Nd-4.2; 1.6/2.5; 56%		Nd-14; 3.1/2.0; 80%	Nd-2.7; 0.11/0; 4%	[7]

Taihu Lake (29)	6.51-64.5;0.187/-;100%	5.05-334;0.099/-;100%	5.08-259;2.41/-;100%	Nd-0.249;0.993/-;48.3%		[8]
Marine water						
Dalian (14)		0.17-4.51; 0.94/0.62; -	2.78-5.71; 4.04/3.88; -	0.24-1.09; 0.69/0.67; -		[9]
Bohai Sea	2.54-16.69; 7.02/5.50; -	<MLD; <MLD/<MLD; -				
Yellow Sea	1.48-1.69; 3.68/2.71; -	<MLD-7.65; 14.48/13.55; -				[11]
East China Sea	<MLD; <MLD/ <MLD; -	<MLD; <MLD/<MLD; -				
Nanning	-; 20/-; 92%	-; 4.0/-; 92%	-; 3.0/-; 92%	-; 0.07/-; 83%		[10]
	-; 5.9/-; 100%	-; 6.1/-; 100%	-; 1.3/-; 67%	-; 0.17/-; 83%		
Drink water						
Weifang (8)		3.54-15.92; 7.55/6.60; 100%				[13]
Weifang (4)		0.82-1.19; 1.00/0.99;100%				
Sewage treatment plant (STP) influent waste water						
Pearl River Delta		71.0	23.4	LOQ		[14]
		2248	311	Nd		
		38.4	Nd	Nd		
		166	Nd	Nd		
		35.6	30.5	27.3		
		22.5	Nd	LOQ		
		Nd	LOQ	10.2		
		54.5	79.4	24.7		
Shanghai	13.65	50.78			1477.00	
	24.46	55.35			210.89	
	10.32	62.48			44.15	
	19.14	95.34			77.00	
	18.2	81.16			90.33	
	20.15	76.34			47.15	
	16.89	77.14			1266.67	

Sewage treatment plant (STP) influent waste water								
Pearl River Delta				40.1	11.9	LOQ		
				2541	23.1	Nd		
				32.3	18.4	Nd		
				LOQ	Nd	Nd		
				LOQ	11.4	Nd		
				3.61	4.64	Nd		
				Nd	Nd	Nd		
				38.0	13.6	Nd		[14]
Shanghai	35.61			34.77			50.14	
	35.38			74.05			149.48	
	16.87			40.11			38.79	
	18.71			28.37			84.77	
	12.71			23.64			54.18	
	40.56			29.83			17.00	
	19.45			15.78			159.58	
Snow								
Nanjing		19.0-75.9; 41.7/30.8; 100%	19.3-77.1; 42.4/31.3; 100%	11.9-85.6; 48.3/47.2; 100%		12.1-219.0; 62.3/25.7; 100%	3.0-173.0; 76.8/59.0; 100%	[16]

The arrangement and detailed descriptions of the matrices are in accordance with Table S1 without specification. The full names for OPEs are summarized in Table 1. -:data not available. nd: not detected.

Table S3. Comparison of concentrations (range; arithmetic mean/median; ng/L) of the total organophosphate esters and the predominant Aryl phosphate in water from various water environments in China.

Location	o-TTP/ m-TTP/ p-TTP	TCrP	CDPP	TMPP	TPHP	EHDPP	Reference
River Water							
Shanghai (28) urban					1.67-47.7; 10.3/6.31; 100%		[1]
Shanghai (28) rural					5.03-34.3; 14.5/12.6; 100%		
Beijing (340)		<LOD-4.29;0.18/<LOD;37.5 %	<LOD-4.85;0.43/0.03;48.5 %		<LOD-96.3;4.49/1.31;74.8 %	<LOD-40.3;0.38/<LOD;6.7 %	[2]
Chengdu					47.72-164.81;-/-;100%		[3]
Pearl river					2.74-58; 23.46/21.35; -		[4]
Dongjiang River					4.73-100; 32.66/27.35; -		
Yangtze river basin	<LOD; <LOD/<LOD;0				<LOD; <LOD/<LOD; 0	<LOD-22.1; 11.9/11.5; 87.5%	[5]
	<LOD; <LOD/<LOD;0				<LOD; <LOD/<LOD; 0	<LOD-1.83; 1.83/1.83; 1.1%	

Yi River (1)		1.39		0.26	1.09	[6]
Fangting River (1)		3.34		0.64	2.18	
Lake water						
Luoma Lake (14)		0.71-54.6;<br>6.36/2.13;100%		0.15-8.16;<br>1.80/0.86;<br>100%	<2-3.39;<br>1.86/1.90;<br>88%	[6]
Taihu Lake（25）		Nd-1.5;<br>8.8×10^{-2}/0；<br>12%		Nd-14;<br>1.8/0.97;<br>84%	0.88-12；<br>2.8/2.2；<br>100%	[7]
Taihu Lake (29)			Nd-0.997;<br>0.007/-;<br>55.2%	Nd-97.5;<br>0.145/-;<br>37.9%	Nd-0.252;<br>0.031/-;<br>13.8%	[8]
Marine water						
Dalian (14)			0.11-0.56;<br>0.31/0.2	0.49-2.51;<br>0.85/0.59	0.08-0.5;<br>0.31/0.33	[9]
Bohai Sea				0.07-3.14;<br>0.578/0.16;<br>-		
Yellow Sea				0.05-0.43;<br>0.20/0.15;<br>-		[11]
East China Sea						
Nanning			-;<br>7.5/-;<br>100%	-;<br>1.0/-;<br>100%		[10]
			-;<br>0.07/-;<br>42%	-;<br>0.01/-;<br>33%		
Drink water						
Weifang (8)	Nd-8.97;<br>1.21/nd;<br>37.5%/<br>Nd-5.45;<br>0.80/0.14;<br>87.5%/<br>Nd-1.75;<br>0.25/0.05;<br>62.5%			0.88-7.96;<br>1.34/0.37;<br>100%		[13]
Weifang (4)	Nd-0.52;<br>0.13/nd;<br>25%<br>0.10-0.28;<br>0.15/0.11;<br>100%/<br>Nd-0.07;<br>0.02/nd;<br>25%			0.83-5.91;<br>4.20/5.03;<br>100%		
Sewage treatment plant (STP) influent waste water						

Pearl River Delta			8.25	40.9		[14]
			LOQ	43.5		
			Nd	274		
			Nd	126		
			27.3	20.6		
			LOQ	10.1		
			10.2	290		
			24.7	10.2		
Shanghai				8.99		
				11.09		
				46.09		
				44.73		
				49.85		
				10.46		
				58.46		
Sewage treatment plant (STP) effluent waste water						
Hebei Hengshui				0.99-4.4;<br>2.4/2.5		[17]
Pearl River Delta			LOQ	108		[14]
			Nd	5.40		
			Nd	2.64		
			Nd	15.1		
			2.49	6.5		
			LOQ	1.38		
			LOQ	1.01		
			3.20	2.94		
Shanghai				9.46		
				9.05		
				44.60		
				38.61		
				4.08		
				6.01		
				45.11		
Snow						
Nanjing				7.2-83.3;<br>23.4/15.2;<br>100%	Nd-3.0;<br>3.0/3.0;<br>14.3%	[16]

The arrangement and detailed descriptions of the matrices are in accordance with Table S1 without specification. The full names for OPEs are summarized in Table 1. -:data not available. nd: not detected.

Table S4. Comparison of concentrations (range; arithmetic mean/median; ng/g) of the total organophosphate esters (OPEs) and the predominant chloroalkyl phosphates in sediment and sludge from various sediment environments in China.

Location	Description of matrix (sample year)	∑OPEs (the total number)	TCEP	TCIPP	TDCPP	Reference
River						
Liao River (24)	(Jun 2018)	19.7-234;64.2/52.3; (13)	1.15-30.1;3.63/1.55; 100%	0.44-13.3;2.98/2.33; 100%	0.48-27.7;4.00/0.93; 100%	[18]
Chengdu	Jinjiang	25.52-296.00; -/-; (7)	3.95-17.25; -/-; 100%	4.10-67.71; -/-; 100%	7.06-7.39; -/-; 28.57%	[3]
Lake						

Luoma Lake (6)	Include the estuary and lake outlet (Nov 2016)	0.04-35.9; 21.0/28.7; (12)	0.01-1.72; 0.38/0.17; 100%	<-0.11; 0.06/0.07; 67%	<-0.03; 0.01/0.02; 67%	[6]
Taihu Lake	Meiliang Bay Gonghu Bay Xukou Bay	5.23-9.01; 7.116/7.175; (7)	1.01-3.17; 1.776/1.535; -	1.07-1.91; 1.466/1.495; -	0.75-1.3; 0.991/0.97; -	[19]
Taihu lake（23）	Taihu Lake and its tributaries (Nov 2016)	8.1-4.2×10²；97/-；（12）	Nd-28; 3.0/1.8; 56%	0.27-10; 1.2/0.45; 100%	Nd-2.2; 9.5×10⁻²/0；4.3%	[7]
			Sea area			
Taiwan Strait (32)		5263-34232; 12796/-; (10), pg/g	LOD-4232; 1096/-; 100%, pg/g	442-3448; 11314/-; 100%, pg/g	LOD-206; 124/-; 100%, pg/g	[20]
Beibu Gulf	Ponds and	4.35-22.1; -/7.75; (11)				[12]
	open areas	4.51-11.7; -/7.42; (11)				
Bohai Sea and vicinity (48)	Urbanized and industrializing areas (2012 and 2016)	1.76-49.9; 12.7/9.13; (9)	Nd-14.8; 4.36/3.67; 58.3%	Nd-17.9; 5.37/3.82; 91.7%	-	
Northern east China (43)	Urbanized and industrializing areas (2011)	8.58-169; 41.6/31.6; (10)	Nd-14.5; 4.99/4.17; 37.2%	Nd-39.8; 6.95/5.37; 97.7%	Nd-4.44; 4.44/4.44; 2.33%	[21]
Northern Chinese coastal waters (91)	Urbanized and industrializing areas (2011, 2012 and 2016)	1.76-169; 26.3/20.3; (11)	Nd-14.8; 4.59/3.77; 48.4%	Nd-39.8; 6.14/4.58; 94.5%	Nd-4.44; 4.44/4.44; 1.10%	
			Sewage treatment plant (STP) influent waste water			
Henan (24)	(Jan-Mar 2015）					
			Sewage treatment plant (STP) effluent waste water			
Hebei Hengshui (6)	Manufacturing plant (0-10cm) (Dec 2016)		61-79000; 29000/22000；-	280-190000; 22000/29000；-		[17]
Sludge						
The pearl river delta	Industrial wastewater	96.7-88.1; 482.4/482.4;	6.9-7.1; 7/7;	6.3-7.6; 6.95/6.95; -	11.8-15; 13.4/13.4; -	[21]
The pearl river delta	Domestic wastewater	100.9-290.2; 195.41/184.9;	8.1-15.2; 11.09/10;	7.9-35; 15.8/13.1;	12.6-64; 21.84/16.9;	[22]

The pearl river delta	Combined wastewater	265.6-1312.9; 657.25/532.55;	- 9.8-17.1; 13.875/14.15; -	- 12-54.4; 29.2/24.8; -	- 14.6-41.5; 27.74/25.95; -	[22]

The full names for OPEs are summarized in Table 1. -:data not available. nd: not detected.

Table S5. Comparison of concentrations (range; arithmetic mean/median; ng/g) of the total organophosphate esters (OPEs) and the predominant Alkyl phosphate in sediment from various sediment environments in China.

Location	TEP	TPP	TNBP	TBOEP	TDBPP	TEHP	TiBP	TMP	TBEP	Reference
River										
Liao River (24)	0.56-11.4; 2.89/1.80; 100%	0.78-12.6; 2.75/1.40; 100%	2.88-49.1;16.0/6.95;100%	1.11-69.0; 11.3/2.73; 100%		1.36-20.2; 4.99/2.44; 100%	0.30-12.7; 3.79/3.33; 100%			[18]
Chengdu			4.66-31.36; -/-; 100%			4.78-7.66; -/-; 100%			64.46-225.03; -/-; 85.71%	[3]
Lake										
Luoma Lake (6)	<-34.5; 19.7/25.9; 67%		<-0.05; 0.02/0.02; 67%		<-0.03; 0.01/<;33%	0.003-0.16; 0.05/0.04; 100%		<2.14; 0.71/0.67; 67%	<-0.001; 0.001/0.001; 67%	[6]
Taihu Lake		<0.2; <0.2/<0.2; 0							1.38-2.32; 1.713/1.615;-	[19]
Taihu lake（23）	Nd-2.8; 0.37/0; 43%		Nd		Nd			Nd	Nd	
Sea area										
Taiwan Strait (32)	Nd		2994-15332; 7916/-; 100%, pg/g	Nd-634; 343/-; 62.5%, pg/g		91-16803; 1821/-; 100%, pg/g				[20]
Bohai Sea and vicinity (48)	nd-0.512; 0.303/0.295; 56.3%		Nd-13.1; 2.39/1.78; 60.4%	Nd-11.9; 1.85/1.20; 60.4%			Nd-5.69; 1.74/1.47; 81.3%	Nd-7.77; 2.46/0.842; 12.5%		
Northern east China (43)	0.207-3.59; 0.711/0.539; 100%	nd-2.42; 0.934/0.458; 9.30%	Nd-25.7; 5.51/4.54; 95.3%	Nd-8.03; 1.84/1.53; 60.5%			3.60-125; 22.7/16.7; 100%	Nd-0.967;0.474/0.397;18.6%		[21]
Northern Chinese coastal waters (91)	Nd-3.59; 0.553/0.393; 76.9%	nd-2.42; 0.934/0.458;	Nd-25.7; 4.21/3.22; 76.9%	Nd-11.9; 1.84/1.25; 60.4%			Nd-125; 12.7/5.50; 90.1%	Nd-7.77; 1.33/0.461;		

	4.40%	15.4%		
Sludge				
The pearl river delta	9.9-10.6; 10.25/10.25;		25.1-25.8; 25.45/25.45;	
The pearl river delta	5.2-13.6; 10.01/10.4;		26.2-169.2; 81.367/636;	[22]
The pearl river delta	8.4-265; 74.11/47.3;		11.8-165.1; 53.5/28;	

The arrangement and detailed descriptions of the matrices are in accordance with Table S4 without specification. The full names for OPEs are summarized in Table 1. -:data not available. nd: not detected.

Table S6. Comparison of concentrations (range; arithmetic mean/median; ng/m^3) of the total organophosphate esters (OPEs) and the predominant Aryl phosphate in sediment from various sediment environments in China.

Location	TCrP	CDPP	TMPP	TPHP	EHDPP	Reference
			River			
Liao River (24)			0.53-11.1; 2.26/1.31; 100%	0.55-6.35; 2.13/1.76; 100%	1.19-16.4; 3.35/2.71; 100%	[18]
Chengdu				8.36-14.10; -/-; 71.43%		[3]
Lake River						
Luoma Lake	0.04-0.1; 0.06/0.06; 67%			<-0.03; 0.01/0.01; 83%	<-0.14; 0.06/0.06; 67%	[23]
Taihu Lake				0.4-1.01; 0.61125/0.57; -		[19]
Taihu lake（23）	Nd-0.55; 9.2×10^{-2}/0；22%			Nd-55； 6.6/1.7； 83%	Nd-0.94; 0.38/0.32; 78%	[7]
			Sea water			
Taiwan Strait (32)			Nd-671; 42/-; 93.8%, pg/g	46-4364; 549/-; 100%, pg/g		[20]
Bohai Sea and vicinity (48)				Nd-3.33; 1.74/1.64; 16.7%	Nd-7.28; 5.54/5.20; 8.33%	[21]
Northern east China (43)				Nd-34.5; 4.61/2.58; 62.8%		

Northern Chinese coastal waters (91)	Nd-34.5;<br>3.95/2.30;<br>38.5%	Nd-7.28;<br>5.54/5.20;<br>4.40%	
Sewage treatment plant (STP) effluent waste water			
Hebei Hengshui	Nd-1.5;<br>0.47/0.48<br>-		[17]
The pearl river delta	26.1;<br>26.1/26.1;<br>-		[22]
The pearl river delta	16.3-62.9;<br>36.94/37.1;<br>-		[22]
The pearl river delta	30.2-656.7;<br>201.16/149.85;<br>-		[22]

The arrangement and detailed descriptions of the matrices are in accordance with Table S4 without specification. The full names for OPEs are summarized in Table 1. -:data not available. nd: not detect.

Table S7. Comparison of concentrations (range; arithmetic mean/median; Detection ratio% ng/m^3) of the total organophosphate esters and the dominant chloroalkyl phosphates in air from various air environments in China.

**Location**	**Description of matrix (sample year)**	**∑OPEs (the total number)**	**TCEP**	**TCIPP**	**TDCPP**	**Reference**
Urban area						
Zhengzhou	High-tech development zone; laboratory building (Jun to Nov2018)	0.30-3.46；<br>1.04/1.36；<br>(6)	0.15-1.71；<br>0.39/0.60；<br>100%	0.04-1.76；<br>0.22/0.41；<br>100%		[24]
Dalian (2)	Bohai Sea and Yellow Sea urban district (Sep 2017)	0.21-0.36;<br>0.29/-<br>(9)	0.091-0.194;<br>0.142/-;<br>-	0.082-0.115;<br>0.098/-;<br>-	0.001-0.003;<br>0.002/-;<br>-	[15]
Nanning	Urban and urban	-;<br>420±330/-;<br>(11)<br>(pg/m^3)	-;<br>140/-;<br>92%<br>(pg/m^3)	-;<br>230/-;<br>100%<br>(pg/m^3)	-;<br>0.15/-;<br>17%<br>(pg/m^3)	[10]
Beijing1	15-20m above the ground level (Apr 2016 and Mar 2017)	174-3300;<br>1160/987;<br>(8) pg/m^3	13-1090；<br>270/150；<br>-, pg/m^3	4205-865；<br>396/340；<br>-, pg/m^3	20.1-114；<br>54.8/47.9；<br>-, pg/m^3	
Beijing2	15-20m above the ground level(Apr 2016 and Mar 2017)	310-3666;<br>1450/1230;<br>(8) pg/m^3	42.9-1670；<br>449/260；<br>-, pg/m^3	83.4-773；<br>313/241；<br>-, pg/m^3	14.5-88.7;<br>52.1/52.8;<br>-, pg/m^3	[15]
Beijing（average）			0.406	1.503	0.0369	

Tianjin	15-20m above the ground level (Apr 2016 and Mar 2017)	674-5250; 2180/1590; (8) pg/m3	96-1370; 432/283; -, pg/m3	159-3190; 1080/920; -, pg/m3	12.5-254; 111/88.6; -, pg/m3	
Shijiazhuang	15-20m above the ground level(Apr 2016 and Mar 2017)	417-3180; 1500/1410 (8) pg/m3	80.3-960; 321/218; -, pg/m3	26.3-1009; 443/426; -, pg/m3	39.1-818; 268/93.8; -, pg/m3	
Baoshan (55)	Sub-urban near the cement and chemical industrial plants and residential areas (2008)	1.8-53.7; 19.4/16.6; (6)	0.2-10.5; 3.1/3.5; 100%	Nd-16.9; 3.8/2.9; 98%	0.03-16.9; 2.3/0.8; 100%	[25]
Xujiahui61()	Urban near the residential and office building (2008)	1.5-54.6; 6.6/4.4; (6)	0.1-10.1; 2.2/1.8; 100%	0.1-9.7; 1.4/1.0; 100%	Nd-23.9; 1.0/0.3; 97%	
Guangdong University of Technology (24)	$PM_{2.5}$ samples (May 2017 to Apr 2018)	4.17-75.2; 15.9/15.2; (11)	0.77-3.61; 1.84/1.69; 100%	0.81-16.6; 4.07/4.13; 100%	0.12-2.76; 0.41/0.32; 100%	
Guangzhou (24)	South China Institute of Environmental Science $PM_{2.5}$(May 2017 to Apr 2018)	4.01-53.1; 13.5/14.0; (11)	0.73-3.77; 1.64/1.75; 100%	0.76-16.0; 3.90/4.27; 100%	0.12-1.09; 0.38/0.36; 100%	[26]
Taiyuan (24)	Shanxi University $PM_{2.5}$(May 2017 to Apr 2018)	3.10-544; 19.5/19.4; (11)	0.46-183; 4.11/2.73; 100%	0.56-9.24; 2.10/2.30; 100%	0.03-20.4; 0.38/0.33; 100%	
			Remote area			
Rural of Beijing l	The northern east of Beijing 15-20m above the ground level (Apr 2016 and Mar 2017)	139-1508; 531/457; (8) pg/m3	5.22-146; 51.4/50.4; - pg/m3	80.8-1070; 318/209; - pg/m3	1.73-36.5; 17.2/13.2; - pg/m3	[15]
			Sea area			
Bohai and Yellow Seas	(Jun and Jul 2016)	2.3-270; 150/170; （9）	Nd-73; 38/38; 100%, pg/m3	Nd-130; 45/42; 100%, pg/m3	Nd-4.3; 0.81/0.57; 100%, pg/m3	[27]

North Huangcheng Island	(Jun and Jul 2016)	1.2-360; 47/31; (9)	0.69-120; 14/10; 100%, pg/m³	0.69-120; 14/10; 100%, pg/m³	Nd-2.6; 0.35/Nd; 100%, pg/m³	
Northern South China Sea	(Sep-Oct 2013)	47.1-160.9; 92.9/90.4; (8), pg/m³	14-107; 46.1/43.5; -, pg/m³	15-38; 24.6/25; -, pg/m³	1.3-4.5; 2.64/2.2; -, pg/m³	[28]
Beijing (15)	Dormitories, residential homes and offices (2014-015)	1.0-20; 5.2/4.7; (14)	0.063-3.8; 0.50/0.17; 100%	0.28-14; 3.8/3.8; 100%	Nd-0.025; 0.0038/Nd; 20%	[29]
Harbin	Dayside bedroom	1.8-460; 35/9.1; 100% (12)	0.16-60; 5.2/0.89; 100%	Nd-390; 24/3.9; 97.2%	Nd-2.66; 0.48/0.25; 77.8%	
	Nightside bedroom	0.41-160; 27/15; 100% (12)	Nd-7.8; 1.0/0.75; 94.7%	Nd-80; 15/12; 97.4%	Nd-1.6; 0.38/0.25; 84.2%	[30]
	Living room	6.8-940; 130/59; 100% (12)	Nd-15; 3.7/2.9; 96.7%	3.4-930; 120/54; 100%	Nd-2.6; 0.60/0.55; 80.0%	

The full names for OPEs are summarized in Table 1. -:data not available. nd: not detected.

Table S8. Comparison of concentrations (range; arithmetic mean/median; Detection ratio% ng/m³) of the total organo-phosphate esters and the predominant Alkyl phosphates in air from various air environments in China.

Location	TEP	TPP	TNBP	TiBP	TBOEP	TEHP	TMP	TBEP	Reference
					Urban area				
Zhengzhou			0.03-0.42; 0.14/0.17 100%					<0.0002-0.16; 0.01/0.03 83%	[24]
Dalian (2)			0.025-0.025; 0.025/-		0.004-0.006; 0.005/-; -	Nd-nd; nd/-; -			[15]
Nanning			-; 18/-; 75% (pg/m³)	-; 11/-; 100% (pg/m³)	-; 20/-; 75% (pg/m³)	-; 0.09/-; 8% (pg/m³)			[10]
Beijing1								0.08-266; 65.3/63.1; -, pg/m³	
Beijing2								9.99-937; 143/47.6; -, pg/m³	
Beijing (average)								0.0708	[15]
Tianjin								5.68-134; 44.2/25.6; -, pg/m³	
Shijiazhuang								1.82-625; 106/57.3; -, pg/m³	
Baoshan (55)								Nd-6.6; 0.1/Nd;	[25]

								4%	
Xujiahui (61)								Nd-0.7; 0.01/Nd; 2%	
Guangdong University of Technology (24)	0.19-1.29; 0.55/0.59; 100%		0.26-3.99; 1.42/1.37; 100%	0.23-8.84; 1.52/1.63; 100%	Nd-0.82; 0.10/0.09; 34.8%	0.42-5.95; 0.96/0.73; 100%			
Guangzhou (24)	0.21-1.70; 0.53/0.59; 100%		0.32-3.06; 1.02/1.10; 100%	0.24-9.56; 0.88/0.99; 100%	Nd-0.71; 0.05/0.07; 50%	0.31-2.17; 0.70/0.63; 100%			[26]
Taiyuan (24)	0.62-13.3; 1.64/1.55; 100%		0.29-4.71; 1.17/1.35; 100%	0.44-182; 2.65/2.12; 100%	0.06-2.12; 0.44/0.45;100%	0.11-39.0; 1.02/0.67; 100%			
Remote area									
Rural of Beijing								7.93-61.4; 28.2/21.3; -, pg/m^3	[15]
Sea area									
Bohai and Yellow Seas			0.46-36; 8.9/8.0; 100%pg/m^3	0.30-170; 34/30; 100%pg/m^3		Nd-5.3; 1.6/1.0; 93%, pg/m^3			[27]
North Huangcheng Island			Nd-190; 11/2.7; 100% pg/m^3	Nd-82; 8.1/4.8; 100%, pg/m^3		Nd-30; 2.3/1.1; 93% pg/m^3			
Northern South China Sea			1.4-4.8; 2.73/2.5; -, pg/m^3	1.1-3.8; 2.25/2.1; -, pg/m^3		2.3-16; 5.02/3.6; -, pg/m^3			[28]
Beijing (15)	0.20-0.83; 0.47/0.39; 100%		0.063-0.40; 0.18/0.17; 100%	0.069-0.33; 0.17/0.15; 100%		Nd; Nd/Nd; -	Nd-0.14; 0.017/Nd; 33%	0.0014-0.013; 0.0043/0.0027; 100%	[29]
Harbin		Nd-0.36; 0.09/0.05; 97.2%	0.06-0.96; 0.47/0.49; 100%	0.11-5.6; 1.9/1.8; 100%	Nd-62; 2.1/nd; 13.9%	Nd-0.51; 0.15/0.11; 91.7%			
		Nd-0.26; 0.07/0.06; 92.1%	0.01-1.4; 0.44/0.40; 100%	Nd-3.4; 0.97/0.58; 97.4%	Nd-70; 3.8/nd; 13.2%	Nd-2.9; 0.17/0.04; 71.1%			[30]
		Nd-0.27; 0.06/0.03; 86.7%	Nd-5.3; 0.51/0.21; 100%	0.02-1.8; 0.45/0.34; 100%	Nd-9.7; 0.37/nd; 6.7%	Nd-0.31; 0.08/0.05; 93.3%			

The arrangement and detailed descriptions of the matrices are in accordance with Table S10 without specification. The full names for OPEs are summarized in Table 1. -:data not available. nd: not detected.

Table S9. Comparison of concentrations(range; arithmetic mean/median; Detection ratio% ng/m^3) of the total organophosphate esters(OPEs) and the predominant Aryl phosphate in air from various air environments in China.

Location	TCrP	CDPP	TMPP	TPHP	EHDPP	Reference
Urban area						
Zhengzhou	<0.0137-0.18; 0.02/0.05;			0.03-0.36; 0.09/0.11;		[24]

	87%			100%		
Dalian (2)			0.001-0.005; 0.003/-		0.003-0.003; 0.003/-; -	[15]
Nanning			-; 0.37/-; 25% (pg/m^3)	-; 0.56/-; 17% (pg/m^3)		[10]
Beijing1					32.3-216; 102/99.6; -, pg/m^3	
Bejing2					39.4-418; 156/106; -, pg/m^3	
Beijing（average）					0.091	[15]
Tianjin					41.6-2516; 113/101; -, pg/m^3	
Shijiazhuang					45.3-338; 147/156; -, pg/m^3	
Baoshan（55）				0.2-32.0；7.2/5.9；100%		[25]
Xujiahu（61）				0.06-14.0；1.3/0.5；100%		
Guangdong University of Technology (24)	0.51-20.6; 2.27/1.90; 100%			0.33-5.18; 1.71/1.81; 100%	0.31-5.56; 1.03/0.91; 100%	[26]
Guangzhou (24)	0.55-8.18; 2.13/2.12; 100%			0.37-4.32; 1.54/1.40; 100%	0.40-2.53; 0.77/0.70; 100%	
Taiyuan (24)	0.3-33.7; 4.59/6.72; 100%			0.13-49.4; 0.98/0.72; 100%	0.07-7.39; 0.47/0.47; 100%	
Remote area						
Rural of Beijing					10.4-47; 28.4/25.3; -, pg/m^3	[15]
Sea area						
Bohai and Yellow Seas				0.19-4.7; 2.0/1.7; 100%, pg/m^3		[27]
North Huangcheng Island				0.17-22; 4.4/2.7; 100%, pg/m^3		
Beijing (15)	Nd-0.0019; 0.00029/Nd; 47%	Nd-0.013; 0.0023/0.0010; 67%		0.0091-0.43; 0.078/0.034; 100%	0.0028-0.056; 0.015/0.084; 100%	[29]

Haibin			0.01-7.3; 0.28/0.06; 100%	Nd-0.12; 0.02/nd; 19.4%	
			Nd-138; 4.6/0.04; 97.4%	Nd-0.05; 0.01/nd; 23.7%	[30]
			0.01-1.1; 0.20/0.12; 100%	Nd-0.07; 0.01/nd; 30.0%	

The arrangement and detailed descriptions of the matrices are in accordance with Table S10 without specification. The full names for OPEs are summarized in Table 1. -:data not available. nd: not detected.

Table S10. Comparison of concentrations (range; arithmetic mean/median; detection ratio ng/g) of the total organophosphate esters (∑OPEs) and the predominant chlorinated phosphates in dust from various dust environments in China.

Location	Description of matrix (sample year)	∑OPEs (the total number)	TCEP	TCIPP	TDCPP	Reference
			Outdoor			
Beijing road (65)	Road（Dec-Nov2014）	278-11293;1861/933; (14)	<5-4185; 397/247; 86.2%	57.1-6142; 1010/384; 100%	<16-233;37.2/30.7; 78.5%	[31]
Tianjin (17)	Open recycling sites for outdoor recycling areas	1390-42700; 11500/6590; (12)	161-9740; 2330/1687; 100%	50.7-16300; 4280/1516; 100%	Nd-4600; 587/265; 95%	[32]
Tianjin (5)	Semi-closed recycling sites for workshop areas	914-7940; 3630/2690; (12)	<91.2-2150; 726/411; 100%	39.9-1270; 508/250; 100%	62.5-599; 312/336; 95%	
Nanning	Dustfall	-; 2.5±3.7/-; (11)	-; 0.39/-; 100%	-; 1.7/-; 100%	-; 0.001/-; 8%	[10]
Henan street (60)	Urban street (Jul 2018)	2.77-505; 92.1/59.3; (8)	0.45-129; 23.2/13.2; 100%	0.68-097; 33.9/20.4; 100%	-	[33]
Nanjing(urban)(8)	Subway entrance of heavy transportation and chemical industry park (Oct 2017)	66.79-367.01; 174.39/157.25; (13)	3.21-15.35; 7.81/6.85; 100%	29.61-216.77; 90.65/81.99; 100%	1.17-3.46; 2.61/2.77; 100%	[34]
Nanjing (rural)(10)	Countryside and kindergarten and school (Mar 2016, Dec 2016, Jul 2017, Oct 2017)	10.89-31.27; 20.99/20.08; (13)	0.57-12.77; 3.25/1.85; 100%	3.21-16.16; 7.14/4.12; 100%	0.45-1.50; 0.88/0.80; 100%	[34]
Chongqing (37)	Main road dust (Jul 2016)	114-1600; -/292; （12）	1.04-103; -/16.4; 100%	Nd-715; -/112; 97%	Nd-209; -/6.59; 75.8%	[35]

Chongqing (6)	Industrial road dust (Jul 2016)	103-967; -/476; （12）	Nd-75.5; -/29.9; 83.3%	Nd-46.1; -/7.94; 50%	Nd-149; -/58.1; 67%	
Chongqing (19)	Campus walking street dust (Jul 2016)	3.69-870; -/48.8; （12）	Nd-16.0; -/4.26; 78.9%	Nd-43.9; -/5.88; 89.5%	Nd-1.44; -/1.44; 5.3%	
Chongqing (28)	Campus road dust（Jul 2016）	9.15-840; -/203; （12）	Nd-75.6; -/4.38; 96.4%	Nd-109; -/18.9; 89.3%	Nd-41.0; -/16.2; 21.4%	
Chengdu street (31)	Street dust (Apr 2014)	94-1484.6; 512.94/347.89; (7)	7.8-402.3; 87.88/55; 100%	5.4-278.3; 93.87/44.4; 100%	Nd-81.8; 17/7.7; 74.2%	[36]
			Indoor			
Beijing (13)	Dormitory indoor (Feb-Mar, Jul-Aug2015)	1.5-18; 6.9/4.3; （14）ug/g	0.30-3.6; 1.1/0.81; 100%, ug/g	0.63-6.2; 1.9/1.3; 100%, ug/g	Nd-2.1; 0.24/nd; 46%, ug/g	
Beijing (39)	Residential home (Feb-Mar, Jul-Aug2015)	1.6-16; 5.9/5.9; （14）,ug/g	0.13-3.0; 0.97/0.79; 100%, ug/g	0.26-13; 1.8/1.4; 100%	Nd-0.93; 0.16/0.12; 67%, ug/g	[29]
Beijing (49)	Office (Feb-Mar, Jul-Aug2015)	2.8-40; 14/11; （14）,ug/g	0.85-14; 4.5/3.7; 100%, ug/g	0.33-19; 4.5/3.1; 100%, ug/g	Nd-3.1; 0.49/0.24; 88%, ug/g	
Shenyang urban dormitories (8)	Dormitories (March to May in 2014)	-; 18300/14500; (11)	-; 447/399; 88%	-; 9730/5980; 100%	-; 372/162; 100%	
Baoding urban dormitories (8)	Dormitories (March to May in 2014)	-; 12500/6540; (11)	-; 2360/2000; 100%	-; 8230/3300; 100%	-; 123/3703; 50%	
Harbin urban dormitory (18)	Dormitories (March to May in 2014)	-; 10100/6190; (11)	-; 1260/850; 100%	-; 4310/2960; 100%	-; 383/175; 89%	[37]
Harbin urban home (15)	Home (March to May in 2014)	-; 20400/7150; (11)	-; 2020/1140; 100%	-; 3790/2290; 100%	-; 692/502; 93%	
Public in Harbin (18)	Home (March to May in 2014)	-; 11300/5340; (11)	-; 5450/1720; 100%	-; 1410/1170; 100%	-; 1310/195; 89%	
College libraries in Xining	College libraries (Oct-Dec2017)		110-7719; -/2584; 100%	54-2336; -/499; 100%	43-3827; -/656; 100%	
College libraries in Lanzhou	College libraries (Oct-Dec2017)		109-10153; -/1749; 100%	1007-39310; -/3046; 100%	59-9331; -/1801; 100%	
College libraries in Xi’an	College libraries (Oct-Dec2017)		100.8-21782; -/579; 95.6%	1699-48321; -/6148; 100%	212-11873; -/2934; 100%	[32]
College libraries in Dalian	College libraries (Oct-Dec2017)		Nd-2843; -/579; 95.6%	2547-13187; -/3341; 100%	400-5137; -/1008; 100%	

College libraries in Changchun	College libraries (Oct-Dec2017)		57-1150; -/426; 100%	1003-19504; -/5419; 100%	100-2086; -/279; 100%	
College libraries in Harbin	College libraries (Oct-Dec2017)		10.6-4125; -/1013; 100%	617-200393; -/4258; 100%	53.3-3588; -/357; 100%	
College libraries in Chengdu	College libraries (Oct-Dec2017)		546-44321; -/3547; 100%	119-40599; -/2378; 100%	1816-33367; -/2994; 100%	
College libraries in Beijing	College libraries (Oct-Dec2017)		3817-41263; -/9758; 100%	2624-30194; -/4157; 100%	379-3989; -/1167; 100%	
College libraries in Baoding	College libraries (Oct-Dec2017)		596-4329;-/1879;100%	372-33715; -/5488;100%	Nd-252; -/97.2; 83.7%	
College libraries in Zhengzhou	College libraries (Oct-Dec2017)		2217-34174; -/4754; 100%	1496-28647; -/3319; 100%	1832-15969; -/2634;100%	
College libraries in Tsinghua	College libraries (Oct-Dec2017)		100-33521; -/3426; 100%	279-28174; -/2169; 100%	23.7-1314; -/504; 100%	
College libraries in Shanghai	College libraries (Oct-Dec2017)		3421-68384; -/7658; 100%	5869-64952; -/9083; 100%	428-5352; -/1587; 100%	
College libraries in Guangzhou	College libraries (Oct-Dec2017)		3892-45873;-/8644; 100%	569-8779;-/2043; 100%	483-5437; -/1013; 100%	
Guangzhou (45)	Residential house (May 2015 to July 2017)	1042-29900; 80000/4798;(8)	176-19511; 2770/712; -	375-3847; 970/555; -	27.0-1735; 352/131; -	
Guangzhou (45)	Office（May 2015 to July 2017)	726-16652; 3935/5241;(8)	105-5199; 1158/552; -	192-8486; 2607/1719; -	57.5-1038; 247/192; -	
Guangzhou (45)	Chemical laboratory（May 2015 to July 2017)	1436-3179; 2205/2000 (8)	312-980; 629/597; -	321-938; 601/546; -	112-412; 214/118; -	[38]
Guangzhou (45)	Instrumental house（May 2015 to July 2017)	8667-39312; 21418/23885;(8)	824-10953; 4734/3733; -	943-34662; 13886/6790; -	232-10747; 2187/980; -	
Indoor dust in China	Living room and bedroom include Shanghai, Beijing, Qiqihar, Nanjing, Xinjiang, Shandong and Guangzhou	149-47400; -/1120;(20)	39.7-45900; -/298; 100%	<0.8-1610; -/29.8; 72%	<1.1-4680; -/37.6; 72%	[39]

(2010-2011)

The full names for OPEs are summarized in Table 1. -:data not available. nd: not detected.

Table S11. Comparison of concentrations (range; arithmetic mean/median; ng/g) of the total organophosphate esters (OPEs) and the predominant Alkyl phosphates in dust from various dust environments in China.

Location	TEP	TPP	TNBP	TiBP	TBOEP	TEHP	TBEP	TMP	Reference
Outdoor									
Beijing road (65)	<0.6-295;53.7/28.6;75.4%		3.80-180;41.1/29.2;100%	<0.6-368;20.3/11.1;95.4%		1.28-19.0;5.97/5.15;100%	3.21-724;73.8/25.7;100%	<1-17.5;3.03/2.30;98.5%	[31]
Tianjin (17)	13.5-520;101/48.7;100%		10.7-53.7;24.4/20.5;100%	<76.5-309;97.3/77.2;100%	Nd-1740;683/544;82%	14.0-13600;1680/524;100%			[40]
Tianjin (5)	14.7-76.4;29.7/18.7;100%		<10.7-58.7;22.9/13.7;100%	<76.5-256;100/79.8;100%	Nd-571;220/Nd;82%	16.5-125;60.3/27.8;100%			
Nanning			-;0.09/-;100%	-;0.29/-;100%	-;nd/-;-	-;0.002/-;17%			[10]
Henan street	0.20-65.7;7.33/2.96;100%	0	<LOD-7.11;1.50/1.08;90%		<LOD-11.0;1.58/0.59;83%			<LOD-9.09;0.89/0.19;50%	[33]
Nanjing(urban) (8)	1.17-7.30;4.56/4.43;100%	Nd-0.11;0.02/Nd;25%	0.85-6.03;2.98/2.82;100%			15.15-39.18;27.21/26.75;100%	1.25-33.18;11.03/5.35;100%	Nd;Nd/Nd;Nd	[34]
Nanjing (rural) (10)	1.04-4.74;2.52/2.49;100%	Nd-0.16;0.02/Nd;30%	Nd-8.66;2.23/1.33;90%			1.02-5.06;2.27/1.64;100%	0.03-0.20;2.27/1.64;100%	Nd-2.20;0.22/Nd;12.5%	
Chongqing (37)	Nd-242;-/4.92;97%		Nd-715;-/112;97%		Nd-11.0;-/2.27;87.9%	Nd-12.8;-/0.88;93.9%		Nd-105;-/1.50;51.5%	[35]
Chongqing (6)	Nd-57.4;-/12.2;67%		Nd-757;-/477;66.7%		Nd-29.0;-/3.78;83.3%	0.60-74.0;-/6.17;100%		Nd-56.4;-/72.1%	
Chongqing (19)	Nd-438;-/4.47;94.7%		Nd-631;-/13.7;89.5%		Nd-7.15;-/2.08;84.2%	Nd-4.77;-/0.84;78.9%		Nd-8.48;-/1.25;84.2%	
Chongqing (28)	Nd-35.0;-/3.95;100%		Nd-470;-/80.9;82.1%		Nd-43.5;-/2.79;92.9%	Nd-12.7;-/1.02;89.3%		Nd-26.8;-/0.50;75%	
Chengdu street (31)			Nd-121;26.18/11.2;83.9%			Nd-489.8;61.37/26;93.5%	63.2-420.3;143.13/118.9;100%		[36]
Indoor									
Beijing (13)	Nd-0.95; 0.093/nd; 23%		Nd-0.57; 0.075/0.020; 77%	Nd-0.049; 0.011/0.0089; 85%		Nd-14; 1.5/0.56; 69%	0.013-0.15; 0.078/0.089; 100%	Nd-0.35; 0.073/nd; 31%	[29]
Beijing (39)	Nd-3.0; 0.34/0.17; 77%		Nd-0.30; 0.038/0.030; 82%	Nd-0.076; 0.017/0.013; 95%		Nd-3.3; 0.49/0.28; 74%	Nd-5.5; 0.35/0.11; 97%	Nd-3.9; 0.45/nd; 46%	
Beijing (49)	Nd-3.3; 0.50/0.089; 61%		Nd-0.88; 0.057/0.026; 78%	Nd-0.091; 0.022/0.012; 90%		Nd-0.98; 0.23/0.22; 67%	0.0087-31; 1.8/0.14; 100%	Nd-2.5; 0.26/nd; 35%	

Shengyang urban dormitories (8)		-；140/116；88%	-;81.1/Nd;25%	-;430/291;100%	-;411/166;50%	-;261/161;100%	
Baoding urban dormitories (8)		-；88.9/75.9；88%	-；92.9/33.2；63%	-；165/Nd;38%	-;730/608;100%	-;181/75.6;100%	
Harbin urban dormitory (18)		-；322/213；94%	-；224/68；67%	-；274/1；78%	-;1100/839;83%	-;230/200;94%	[37]
Harbin urban home (15)		-；316/254；100%	-；487/69.8；60%	-；313/129；67%	-;5030/1520;87%	-;600/376;87%	
Public in Harbin (18)		-；171/11；94%	-；311/172；94%	-；251/137；83%	-;1410/1170;100%	-;157/142;94%	
College libraries in Xining	Nd;-/7.2;0%	Nd-237; -/27.2; 87.6%	Nd-1017; -/119; 90.1%		97-8317; -; -/2843; 100%	Nd; -/6.5; 0%	
College libraries in Lanzhou	Nd-371;-/54.9;87.4%	Nd-559; -/93.7; 84.6%	116-983; -/247; 100%		100-11867; -/3664; 100%	Nd-219; -/28.3; 87.2%	
College libraries in Xi'an	Nd-224; -/20.6; 85.0%	Nd-317; -/48.4; 89.2%	68.4-2184; -/216; 100%		547-10306; -/2839; 100%	Nd-521; -/97.2; 92.6%	
College libraries in Dalian	Nd-217; -/40.7; 88.5%	Nd-317; -/48.3; 82.8%	Nd-3667; -/399; 89.8%		107-10549; -/2389; 100%	Nd-413; -/197; 90.2%	
College libraries in Changchun	Nd-533; -/87.4; 89.8%	Nd-376; -/99.1; 86.4%	34.3-5367; -/724; 100%		238-9911; -/2370; 100%	99.6-2004; -/239; 100%	[32]
College libraries in Harbin	Nd-478; -/97.1; 87.6%	Nd-1357; -/39.5; 80.8%	Nd-1679; -/100; 90.4%		490-7941; -/2986; 100%	Nd-832; -/315; 98.4%	
College libraries in Chengdu	54-2010; -/426; 100%	Nd-107; -/10.6; 81.2%	Nd-217; -/15.9; 80.6%		632-4634; -/3419; 100%	Nd-1000; -/247; 88.5%	
College libraries in Beijing	Nd-577; -/114; 89.3%	Nd-167;-/43.8; 90.6%	Nd-2416; -/348; 88.5%		1142-58176; -/8990; 100%	Nd-1996; -/377; 94.4%	

College libraries in Baoding	Nd-147; -/26.1; 85.2%	Nd-241;-/65.3; 86.4%	Nd-593;-/46.8; 90.8%		379-10036; -/2274; 100%	Nd-881; -/249; 96.6%			
College libraries in Zhengzhou	332-2184; -/567; 100%	Nd-269; -/23.4; 83.8%	219-3267; -/713; 100%		327-7839;-/1012; 100%	Nd-324;-/46.3; 84.5%			
College libraries in Tsingtao	Nd-463; -/29.5; 88.2%	Nd-827; -/68.4; 81.4%	Nd-737; -/54.9; 86.0%		236-19191; -/1294; 100%	Nd-2034; -/199; 82.2%			
College libraries in Shanghai	212-3069; -/934; 100%	Nd-545; -/37.7; 86.5%	Nd-1817; -/186; 85.2%		112-43972; -/6899; 100%	Nd-667; -/80.5; 81.5%			
College libraries in Guangzhou	428-5017; -/11773; 100%	698-5936; -/2126; 100%	100-1013; -/217; 100%		2612-30187; -/5348; 100%	Nd-546; -/20.9; 88.4%			
Guangzhou (45)			LOQ-235; 58.1/21.6; -		LOQ-23526; 2215/88.2; -	Nd-3122; 687/513; -			[38]
Guangzhou (45)			Nd-235; 38.5/26.0; -		LOQ-266; 85.5/59.3; -	83.5-1442; 284/213; -			
Guangzhou (45)			32.9-83.4; 51.2/37.3; -		LOQ-55.5; 34.3/47.5; -	91.3-231; 164/170; -			
Guangzhou (45)			27.4-118; 64.8/58.7; -		LOQ-202;88.8/61.8; -	30.5-926;240/145; -			
Indoor dust in China (50)	<4.9-236; -/13.7; 92%		1.71-306; -/32.2; 100%	1.21-551; -/9.66; 100%	<2-1200; -/45.0; 98%	<8.1-922; -/35.5; 98%		<1.3-4.12;-/<1.3;16%	[31]

The arrangement and detailed descriptions of the matrices are in accordance with Table S13 without specification. The full names for OPEs are summarized in Table 1. -:data not available. nd: not detected.

Table S12. Comparison of concentrations (range; arithmetic mean/median; ng/g) of the total organophosphate esters and the predominant Aryl phosphate in dust from various dust environments in China.

Location	TCrP	CDPP	TMPP	TPHP	EHDPP	Reference
			Outdoor			
Beijing road dust (65)	1.13-2675; 61.6/7.91; 100%	4.04-250; 33.0/19.2; 100%		2.50-1798; 103/65.4; 100%	<9-214; 22.2/10.9; 60%	[31]
Tianjin (17)			146-13600; 1680/524;	42.8-5700; 1530/1150;	Nd-109; 35.5/27.1;	[40]

			100%	100%	95%	
Tianjin (5)			41.4-4110; 945/237; 100%	104-694;348/182;100%	8.80-77.5;30.4/21.1;95%	
Nanning			-; 0.004/-; 25%	-; 0.02/-; 67%		[10]
Henan street (60)	<LOD-32.3; 3.58/1.43; 90%			<LOD-113; 15.0/7.30; 98%		[33]
Nanjing(urban) (8)				4.37-36.57; 17.93/16.59; 100%	0.47-6.12; 3.04/2.14; 100%	[34]
Nanjing (rural) (10)				0.58-7.23; 2.08/1.16; 100%	Nd-0.34; 0.06/Nd; 20%	
Chongqing (37)			Nd-1400;-/14.0; 93.9%	Nd-82.5; -/19.1; 81.8%	Nd-13.9;-/6.10; 36.4%	[35]
Chongqing (6)			0.30-76.4; -/7.10; 100%	Nd-37.9; -/7.77; 50%	Nd-39.6;-/28.2; 33.3%	
Chongqing (19)			Nd-334; -/5.78; 84.2%	Nd-58.2; -/10.1; 84.2%	Nd-10.9; -/4.28; 26.3%	
Chongqing (28)			Nd-268; -/13.2; 82.1%	Nd-234;-/8.21;89.3%	Nd-15.6;-/3.61;42.9%	
Chengdu street (31)				4.9-394.6; 83.55/36.2; 100%		[36]
			Indoor			
Beijing (13)	Nd-0.086; 0.033/0.020; 92%	0.038-4.9; 0.54/0.10; 100%		0.085-3.5; 0.77/0.41; 100%		[29]
Beijing (39)	Nd-0.41; 0.059/0.038; 97%	0.0032-2.2; 0.23/0.0089; 100%		0.067-9.2; 0.68/0.40; 100%		
Beijing (49)	0.0082-0.54; 0.072/0.05; 100%	0.0091-0.54; 0.15/0.11; 100%		0.17-2.7; 0.66/0.61; 100%		
Shengyang urban dormitories (8)				-; 2140/1240; 100%	-; 441/171; 100%	[37]
Baoding urban dormitories (8)				-; 280/182; 100%	-; 179/148; 100%	
Harbin urban dormitory (18)				-; 1720/583; 100%	-; 188/183; 100%	

Harbin urban home (15)		-; 6180/605; 100%	-; 902/257; 100%	
Public in Harbin (18)		-; 617/372; 100%	-; 166/134; 100%	
College libraries in Xining		110-4369; -/1037; 10%	Nd; -/4.1; 0%	
College libraries in Lanzhou		100-11867; -/3664; 100%	Nd-324; -/41.1; 92.5%	
College libraries in Xi'an		341-1331; -/645; 100%	Nd-1111; -/232; 90.8%	
College libraries in Dalian		400-8643; -/1542; 100%	Nd-579; -/131; 88.6%	
College libraries in Changchun		154-9789; -/1419; 100%	200-3671; -/749; 100%	
College libraries in Harbin		103-31841; -/3019; 100%	Nd-1783; -/336; 92.8%	
College libraries in Chengdu		712-31841; -/3019; 100%	Nd-1783; -/336; 92.8%	[32]
College libraries in Beijing		419-3210; -/988; 100%	Nd-2128; -/454; 88.5%	
College libraries in Baoding		300-4386; -/1138; 100%	43.6-257; -/116; 100%	
College libraries in Zhengzhou		274-5487; -/869; 100%	Nd-923 ;-/101; 92.5%	
College libraries in Tsingtao		18.9-3481; -/646; 100%	nd-1718; -/238; 80.8%	
College libraries in Shanghai		58.6-8987; -/2348; 100%	Nd-525; -/97.3; 90.6%	
College libraries in Guangzhou		462-20187; -/1913; 100%	Nd-984; -/68.3; 92.8%	
Guangzhou (45)	7.24-98.1; 45.2/43.7; -	114-1849; 903/906; -		
Guangzhou (45)	8.98-260; 77.5/61.4; -	120-1715; 744/647; -		[38]
Guangzhou (45)	34.4-116;	204-734;		

	82.1/99.7;<br>-	429/347;<br>-		
Guangzhou (45)	29.6-4948;<br>927/57.3;<br>-	200-9779;<br>1758/836;<br>-		
Indoor dust in China (50)	1.75-116;<br>-/13.9;<br>100%	3.52-7620;<br>-/109;<br>100%	1.26-361;<br>-/14.7;<br>100%	[31]

The arrangement and detailed descriptions of the matrices are in accordance with Table S13 without specification. The full names for OPEs are summarized in Table 1. -:data not available. nd: not detected.

Table S13. Comparison of concentrations (range; arithmetic mean/median; ng/g) of the total organophosphate esters and the predominant chloroalkyl phosphates in soil from various soil environments in China.

Location	Description of matrix (sample year)	∑OPEs (the total number)	TCEP	TCIPP	TDCPP	Referenc e
China	Provincial-level administrative away from the point sources (May-Jul2018)	1.70-179;<br>17.5/9.72<br>(12)	0.46-11.8;<br>2.29/1.77;<br>100%	Nd-167;<br>6.62/1.67;<br>57%	Nd-1.87;<br>0.24/0.13;<br>79%	[41]
Hebei Province	Plastic waste recycling in northern China	38-1250；<br>398/-;<br>(9)	7-436;<br>92/-;<br>90%	4-52;<br>21/-;<br>79%	-;<br>-<br>-	[42]
Tianjin (17)	Open recycling sites for outdoor recycling areas	122-2100;<br>829/696;<br>(12)	<3.44-548;<br>114/50.3;<br>100%	34.9-1370;<br>444/378;<br>100%	1.71-177;<br>42.1/23.6;<br>100%	
Tianjin (7)	Semi-closed recycling sites for workshop areas	58.5-316;<br>138/116;<br>(12)	<3.44-23.0;<br>8.52/6.39;<br>100%	5.39-176;<br>55.7/29.1;<br>100%	<0.31-13.7;<br>3.78/2.32;<br>100%	[40]
Tianjin (12)	Farmland around the waste recycling sites	37.7-156;<br>65.2/56.3;<br>(12)	1.72-3.79;<br>2.03/1.72;<br>100%	<2.48-16.8;<br>8.86/8.58;<br>100%	<0.31-28.3;<br>3.89/1.05;<br>100%	
Nanning	Forest soil	-;<br>0.84±0.45/-;<br>(11)	-;<br>0.11/-;<br>83%	-;<br>0.58/-;<br>100%	-;<br>0.007/-;<br>8%	[10]
	Paddy soil	-;<br>2.1±1.4/-;<br>(11)	-;<br>0.13/-;<br>83%	-;<br>1.04/-;<br>100%	-;<br>0.06/-;<br>25%	
Hebei Hengshui (19)	Near the Manufacturing plant (Dec 2016)	Nd-14000;<br>1100/320<br>(ng/kg)	Nd-8600;<br>580/93<br>(ng/kg)	Nd-5300;<br>530/220<br>(ng/kg)		[17]
Central China (31)			0.33-50.6;<br>-/2.93;	0.33-400;<br>-/15.1;<br>-	<0.05-65.7;<br>-/1.24;<br>-	
East/south of China	The urban and rural areas (Mar-Aug2017)		<0.04-137;<br>-/4.22;<br>-	0.82-401;<br>-/38.2;<br>-	<0.05-15.7;<br>-/1.48;<br>-	[43]
Northeast of China			<0.04-56.0;<br>-/2.85;<br>-	3.79-157;<br>-/69.1;<br>-	<0.05-11.6;<br>-/2.13;<br>-	

West of China			<0.04-182; -/1.87; -	<0.12-202; -/9.37; -	<0.05-17.7; -/1.28; -	
Farmland of Three Gorges Reservoir（TGR）(32	Upper reach of the TGR in Chongqing Municipality (Jul 2016)	52.1-680; 266/247; (12)	Nd-3.80; 1.24/1.12; 75%	Nd-19.7; 6.89/5.19; 93.8%	Nd-7.53; 0.26/0.05; 9.37%	[44]
Riparian of Three Gorges Reservoir（TGR）(26)	Upper reach of the TGR in Chongqing Municipality (Jul 2016)	156-1428; 499/365; (12)	Nd-16.1; 2.33/2.03; 92.3%	2.74-130; 13.3/7.2; 100%	Nd-3.45; 0.24/0.05; 30.7%	
Chongqing (14)	Industrial area (Apr-May2017)	25.8-98.3; 55.5/-; (12)	0.29-1.63; 0.94/-; 100%	2.24-16.3; 7.34/-; 100%	0.39-3.04; 1.31/-; 100%	[45]
Chongqing (12)	Residential area (Apr-May2017)	21.5-108; 55.6/-; (12)	0.51-5.43; 2.18/-; 100%	4.68-38.2; 17.6/-; 100%	0.38-2.37; 0.86/-; 100%	
Chongqing (15)	Commercial area (Apr-May2017)	17.7-69.1; 41.8/- (12)	Nd-1.17; 0.60/-; 92.9%	0.75-25.8; 9.08/-; 100%	Nd-4.59; 1.99/-; 85.7%	
Chongqing (15)	City park area (Apr-May2017)	10.7-70.5; 34.0/- (12)	Nd-0.51; 0.29/-; 93.3%	1.75-21.4; 7.82/-; 100%	0.74-12.9; 2.74/-; 100%	
Guangzhou (11)	Park area (Dec 2011)	0.041-0.15; 0.001/0.001 (11)	0.003-0.008; 0.004/0.004; 100%	0.0002-0.003; 0.002/0.001; 100%	0.002-0.015; 0.007/0.005; 100%	[46]
Guangzhou (13)	Paddy/vegetable fields (Dec 2011)	0.063-0.25; 0.12/0.11	Nd-0.047; 0.006/0.003; 53.8%	Nd-0.001; 0.0001/0.0002; 50.7%	Nd-0.011; 0.002/0.003; 38.5%	
Guangzhou (12)	Commercial areas (Dec 2011)	0.25-1.37; 0.46/0.38; (11)	0.030-0.140; 0.093/0.093; 100%	0.001-0.016; 0.006/0.003; 100%	0.005-0.091; 0.034/0.024; 100%,	
Guangzhou (16)	Road greenbelts (Dec 2011)	0.18-0.51; 0.34/0.31; (11)	0.012-0.078; 0.041/0.038; 100%	0.0002-0.005; 0.002/0.003; 100%	0.005-0.035; 0.015/0.014; 100%	
Guangzhou (15)	Residential areas (Dec 2011)	0.11-0.50; 0.23/0.18; (11)	Nd-0.11; 0.018/0.007; 73.3%	Nd-0.014; 0.002/0.001; 73.3%	Nd-0.050; 0.014/0.012; 86.6%	

The full names for OPEs are summarized in Table 1. -:data not available. nd: not detected.

Table S14. Comparison of concentrations (range; arithmetic mean/median; ng/g) of the total organophosphate esters (OPEs) and the predominant Alkyl phosphate in soil from various soil environments in China.

Location	TEP	TPP	TNBP	TMP	TBOEP	TEHP	TBEP	TiBP	Reference
China Provincial-level administrative away	Nd-13.5; 0.75/0.13; 62%	Nd-0.03; 0.002/0.001; 70%	Nd-9.97; 1.01/0.61; 94%	Nd-1.50; 0.03/0.003; 20%	Nd-12.8; 3.41/2.76; 86%	Nd-4.01; 0.62/0.36; 97%			[41]

from the point sources (May-Jul2018)									
Hebei provision			-; 22/-; >60%				20-592; 200/-; 90%	-; 47/-; -	[42]
Tianjin (17)	Nd-13.8; 4.63/3.47; 94%		Nd-3.97; 1.14/1.09; 75%		25.9-306; 62.0/47.3; 100%	0.54-18.5; 4.51/2.87; 100%		2.05-9.56; 4.49/4.89; 100%	
Tianjin (7)	<0.84-1.34; 0.55/0.42; 94%		Nd-2.17; 0.78/1.09; 75%		30.5-72.3; 44.0/36.2; 100%	0.93-12.0; 3.33/1.78; 100%		4.60-7.09; 5.43/5.03; - 100%	[40]
Tianjin (12)	Nd-0.42; 0.38/0.42; 94%		<2.18-4.68; 3.07/3.05; 75%		21.1-59.9; 29.2/28.1; 100%	1.26-45.7; 5.62/1.61; 100%		<4.10-4.59; 2.45/2.05; 100%	
Nanning			-; 0.03/-; 75% -; 0.06/-; 83%		-; 0.01/-; 17% -; 0.10/-; 50%	-; 0.03/-; 25% -; 0.31/-; 75%		-; 0.02/-; 100% -; 0.06/-; 100%	[10]
Hebei Hengshui (19)									
Central of China	<0.06-11.3; -/<0.06 -		0.29-17.6; -/3.06; -	<0.02-5.94; -/<0.02; -	<0.09-12.7; -/0.33; -	<0.10-18.1; -/0.35; - -			
East/south of China	<0.06-18.4; -/<0.06; -		<0.05-10.9; -/3.05; -	<0.02-4.35; -/<0.02; -	<0.09-2.00; -/0.36; -	<0.10-8.28; -/0.50; -			[43]
Northeast of China	<0.06-7.65; -/<0.06; -		1.10-3.83; -/2.96; -	<0.02; -/-; -	<0.09-1.64; -/0.40; -	<0.10-2.14; -/2.85; -			
West of China	<0.06-32.0; -/0.48; -		0.45-14.6; -/1.56; -	<0.02; -/-; -	<0.09-1.31; -/0.35; -	<0.10-5.22; -/0.73; -			
Farmland of Three	Nd-1.11; 0.16/0.08;		Nd-24.4; 3.53/2.44;	Nd-0.38; 0.08/0.03;		Nd-41.6; 7.49/4.42;	Nd-0.556;		[44]

Gorges Reservoir（TGR）(32)	56.3%		96.8%	56.3%		78.1%	0.06/0.10;15.6%	
Riparian of Three Gorges Reservoir（TGR）(32)	Nd-0.565; 0.11/0.01; 50%		0.520-112; 9.16/2.82; 100%	Nd-0.403; 0.12/0.09; 92.3%		Nd-19.4; 6.12/4.48; 88.5%	Nd-1.79; 0.27/0.10; 30.7%	
Residentin Chongqing (12)	Nd-9.17; 5.92/-; 41.7%	2.36-55.8;9.92/-; 100%		3.93-5.03; 4.43/-; 100%		Nd-2.06; 0.54/-; 83.3%	Nd-3.65; 1.79/-; 25%	
Industriala rea in Chongqing (15)	Nd-5.57; 3.05/-; 40.0%	4.31-29.3; 10.7/-; 100%		2.03-7.04; 4.34/-; 100%		0.36-0.98; 0.61/-; 100%	Nd-1.54; 0.94/-; 33.3%	
Commercial area in Chongqing (14)	Nd-7.84; 2.14/-; 21.4%	0.68-5.68; 3.25/-; 100%		0.69-4.48; 3.06/-; 100%		0.19-7.68; 2.70/-; 100%	Nd-3.11; 1.14/-; 33.3%	[45]
City park area in Chongqing (15)	Nd-3.54; 0.77/-; 33.3%	0.85-3.71; 2.02/-; 100%		1.16-3.77; 2.48/-; 100%		Nd-0.58; 0.28/-; 80%	Nd-1.29; 0.54/-; 26.7%	
Park area in Guangzhou (11)	0.004-0.011; 0.006/0.005; 100%		0.002-0.023; 0.009/0.010; 100%	Nd-0.002; 0.0002/0.001; 18.2%	0.013-0.067; 0.046/0.054; 100%	0.001-0.007; 0.002/0.001; 100%		
Paddy/vegetable fields in Guangzhou (13)	0.001-0.007; 0.004/0.004; 100%,		0.002-0.025; 0.015/0.014; 100%	Nd-0.005; 0.001/0.002; 38.5%	0.017-0.114; 0.069/0.068; 100%	Nd-0.015; 0.004/0.003; 61.5%		
Commercial areas in Guangzhou (12)	0.002-0.009; 0.006/0.005; 100%		0.010-0.21; 0.044/0.027; 100%,	Nd-0.001; 0.0002/0.0004; 25%	0.064-0.52; 0.15/0.12; 100%	0.004-0.029; 0.017/0.014; 100%		[46]
Road greenbelts in Guangzhou (16)	0.001-0.008; 0.004/0.004; 100%		0.012-0.057; 0.029/0.024; 100%	Nd-0.001; 0.0001/0.001; 18.8%	0.049-0.25; 0.12/0.11; 100%	0.001-0.006; 0.004/0.004; 100%		
Residential areas in Guangzhou (15)	0.001-0.006; 0.003/0.002; 100%		0.0120-0.046; 0.027/0.026;100%	Nd-0.001; 0.0002/0.0002;33.3%	0.041-0.15; 0.094/0.092;100%	0.001-0.039; 0.006/0.002; 100%		

The arrangement and detailed descriptions of the matrices are in accordance with Table S7 without specification. The full names for OPEs are summarized in Table 1. -:data not available. nd: not detected.

Table S15. Comparison of concentrations (range; arithmetic mean/median; ng/g) of the total organophosphate esters and the predominant Aryl phosphate in soil from various soil environments in China.

Location	TCrP	CDPP	TMPP	TPHP	EHDPP	Reference
China Provincial-level administrative away from the point sources (May-Jul2018)	Nd-115; 2.23/0.58; 52%		Nd-3.48; 0.13/0.04; 79%		Nd-1.23; 0.16/0.13; 94%	[41]
Hebei provision				-; 26/-; >60%	-; 11/-; >60%	[42]
Tianjin (17)			3.03-185; 49.8/46.3; 100%	9.68-303; 100/86.9; 100%	<0.35-4.35; 1.75/1.36; 100%	
Tianjin (7)			0.10-5.99; 2.09/1.84; 100%	1.43-48.4; 14.2/7.02; 100%	0.17-0.81; 0.40/0.18; 100%	[40]
Tianjin (12)			0.87-36.6; 5.08/2.12; 100%	0.53-8.33; 3.00/2.93; 100%	1.12-45.7; 5.62/1.23; 100%	
Nanning			-; 0.003/-; 17% -; 0.11/-; 67%	-; 0.03/-; 17% -; 0.13/-; 67%		[10]
Hebei Heng Shui				Nd-310; 38/23 -		[17]
Central of China			0.05-4.91; -/0.22; -	<0.02-1.70; -/0.05; -	<0.02-1.11; -/0.15; -	
East/south of China			<0.02-5.66; -/0.29; -	<0.02-3.69; -/0.19; -	<0.02-1.29; -/0.16; -	[43]
Northeast of China			0.33-1.36; -/0.68; -	0.22-1.45; -/0.44; -	0.09-0.65; -/0.20; -	
West of China			<0.02-2.66; -/0.30; -	<0.02-1.96; -/0.29; -	<0.02-0.65; -/0.22; -	
Farmland of Three Gorges Reservoir （TGR）(32)			49.9-398; 196/170; 100%	Nd-1.96; 0.23/0.10; 18.8%	Nd-247; 49.2/35.4; 96.9%	[44]
Riparian of Three Gorges Reservoir （TGR）(32)			98.9-1371; 408/249; 100%	Nd-5.54; 0.76/0.10; 30.7%	15.9-126; 57.2/48.2; 100%	

Residential Chongqing (12)			Nd-19.1; 8.06/-; 91.7%	[45]
Industrial area in Chongqing (15)			1.19-9.96; 3.89/-; 10%	
Commercial area in Chongqing (14)			1.13-18.1; 9.86/-; 100%	
City park in Chongqing (15)			Nd-33.6; 8.60/-; 93.3%	
Park area in Guangzhou (11)	Nd-0.019; 0.006/0.012; 45.5%	0.0004-0.004; 0.002/0.002; 100%	0.002-0.009; 0.005/0.004; 100%	[46]
Paddy/vegetab le fields in Guangzhou (13)	Nd-0.048; 0.014/0.013; 76.9%	0.001-0.005; 0.002/0.001; 100%	Nd-0.022; 0.005/0.003; 84.6%	
Commercial areas in Guangzhou (12)	0.009-0.45; 0.071/0.028; 100%	0.005-0.046; 0.023/0.021; 100%	0.006-0.040; 0.020/0.018; 100%	
Road greenbelts in Guangzhou (16)	0.048-0.17; 0.10/0.096; 100%	0.004-0.014; 0.008/0.007; 100%	0.004-0.039; 0.020/0.020; 100%	
Residential areas in Guangzhou (15)	0.020-0.11; 0.051/0.046; 100%	0.002-0.009; 0.004/0.004; 100%	0.001-0.040; 0.009/0.006; 100%	

The arrangement and detailed descriptions of the matrices are in accordance with Table S7 without specification. The full names for OPEs are summarized in Table 1. -:data not available. nd: not detected.

Table S16. Comparison of concentrations (range; arithmetic mean/median; ng/g ww) of the total organophosphate esters and the predominant chloroalkyl phosphates in biota in China.

**Location**	**Description of matrix (sample year)**	**Biota**	**∑OPEs (the total number)**	**TCEP**	**TCIPP**	**TDCPP**	**Reference**
Hebei Heng Shui (8)	near the Manufacturing plant (Dec 2016)	Tree bark(lipid);	5300-19000; 12000/11000;	4200-9100; 6700/4900;	1800-8900; 7500/5700-		[17]
Pearl River Delta	Southern China	Fish (lipid weight）		82.7-4692;-/-	62.7-883;-/-	Nd-251;-/-	[47]
	Qingyuan County	Bird (liquid)		33.7-162;-/-	3.89-21.4;-/-	Nd-43.7;-/-	
Beibu Gulf	Culture ponds; estuaries	Seafood organisms	5.13-39.6; 14.6/-; (11)				[12]
		shrimps	-;12.5/- (11)				

	crabs	-;20.4/-; (11)				
	oysters	-;8.90/-; (11)				
South China Qingyuan[a]	Water snake (7)	1.9±1.2	0.046±0.032	0.31±0.17	0.32±0.78	[48]
	Snake egg (3)	12±2.3	0.16±0.045	0.96±0.18	0.29±0.51	
	Common carp (6)	14±2.4	0.21±0.10	3.1±0.48	0.24±0.21	
Beijing[a]	Topmouth gudgeon		166.88	124.5	22.976	[49]
	Crucian carp		105.72	117.75	13.57	
	Loach		205.53	144.2	32.92	
Longtang Guangdong province[a]	Dragonfly		0.975	1.815		[50]
	Grasshopper		0.545	0.705		
	Cricket adult		1.46	3.50		
	Mole-cricket adult		0.91	2.22		
	Aquatic stinkbug adult		0.39	0.97		
	Terrestrial stinkbug adult		0.28	0.36		
	Terrestrial beetle adult		0.49	1.09		
	Aquatic beetle adult		0.30	1.66		
	Moth		1.54	6.99		

The full names for OPEs are summarized in Table 1. a: only the mean concentration. -: data not available. nd: not detected.

Table S17. Comparison of concentrations (range; arithmetic mean/median; ng/g ww) of the total organophosphate esters (OPEs) and the predominant Alkyl phosphates in biota in China.

Location	TEP	TNBP	TMP	TBOEP	TEHP	Reference
South China, Qingyuan	0.24±0.21	0.79±0.81			0.014±0.011	[48]
	1.0±0.57	7.7±1.4			0.11±0.088	
	0.96±0.40	3.0±1.4			0.13±0.045	
Fish	Nd	43.9-2946	Nd		Nd-3.61	[47]
Bird	Nd	11.7-281	Nd		Nd-13.9	
Beijing		184.08		20.586	115.44	[49]
		228.78		16.6	140.18	
		241.82		24.09	85.67	
Longtang Guangdong province[a]					21.35	[50]
					1.07	
					<LOQ	
					<LOQ	
					1.09	
					15.8	
					<LOQ	
					<LOQ	
					3.35	

The arrangement and detailed descriptions of the matrices are in accordance with Table S16 without specification. a: only the mean concentration. The full names for OPEs are summarized in Table 1. -:data not available. nd: not detected.

Table S18. Comparison of concentrations (range, arithmetic mean ng/g ww) of the total organophosphate esters(OPEs) and the predominant Aryl phosphates in biota in China.

Location	TCrp	CDPP	TMPP	TPHP	EHDPP	Reference
Hebei Heng Shui (8)				Nd-2.3;1.2/1.2<br>-		[17]
South China, Qingyuan				0.23±0.11<br>1.6±1.7<br>6.2±1.8	Nd<br>0.61±0.80<br>0.24±0.32	[48]
Beijing				34.26<br>56.55<br>59.89	20.68<br>21.67<br>33.37	[49]
Long tang Guangdong province				12.515<br>0.585<br>0.26<br>3.12<br>0.17<br>0.28<br><LOQ<br>0.12<br>0.86	0.09<br><LOQ<br>0.22<br><LOQ<br><LOQ<br>0.69<br><LOQ<br><LOQ<br>0.25	[50]

The arrangement and detailed descriptions of the matrices are in accordance with Table S16 without specification. The full names for OPEs are summarized in Table 1. a: only the mean concentration. -:data not available. nd: not detected.

References:

1. Zhang, Z.; Shao, H.; Wu, M.; Zhang, J.; Li, D.; Li, J.; Wang, H.; Shi, W.; Xu, G. Occurrence, Distribution, and Potential Sources of Organophosphate Esters in Urban and Rural Surface Water in Shanghai, China. *Arch Environ Con Tox* **2019**, 77, 115–126, doi:10.1007/s00244-019-00633-w.
2. Shi, Y.; Gao, L.; Li, W.; Wang, Y.; Liu, J.; Cai, Y. Occurrence, distribution and seasonal variation of organophosphate flame retardants and plasticizers in urban surface water in Beijing, China. *Environ Pollut* **2016**, 209, 1–10, doi:10.1016/j.envpol.2015.11.008.
3. Wu D, Yin H, Li S, Wang Z, Deng X, Luo Y, Luo L. Pollution Characteristics of OPEs in the Surface Water and Sediment of Jinjiang River in Chengdu City. Environ. Chem. 2018, 40, 1245–1251 (in Chinese)..
4. Liu, J., He, L., Zeng, X., Yu, Z., Ran, Y., Sheng, G., Fu, J., Occurrence and Distribution of Organophosphorus Flame Retardants/ Plasticizer in Surface Sediments from the Pearl River and Dongjiang River. Asian Journal of Ecotoxicology. **2016**, 11, 436–443 (in Chinese).
5. Xing, L.; Tao, M.; Zhang, Q.; Kong, M.; Sun, J.; Jia, S.; Liu, C. Occurrence, spatial distribution and risk assessment of organophosphate esters in surface water from the lower Yangtze River Basin. *Sci Total Environ* **2020**, 734, 139380, doi:10.1016/j.scitotenv.2020.139380.
6. Xing, L.; Zhang, Q.; Sun, X.; Zhu, H.; Zhang, S.; Xu, H. Occurrence, distribution and risk assessment of organophosphate esters in surface water and sediment from a shallow freshwater Lake, China. *Sci Total Environ* **2018**, 636, 632–640, doi:10.1016/j.scitotenv.2018.04.320.
7. Liu, Y.; Song, N.; Guo, R.; Xu, H.; Zhang, Q.; Han, Z.; Feng, M.; Li, D.; Zhang, S.; Chen, J. Occurrence and partitioning behavior of organophosphate esters in surface water and sediment of a shallow Chinese freshwater lake (Taihu Lake): Implication for eco-toxicity risk. *Chemosphere* **2018**, 202, 255–263, doi:10.1016/j.chemosphere.2018.03.108.
8. Wang, X.; Zhu, L.; Zhong, W.; Yang, L. Partition and source identification of organophosphate esters in the water and sediment of Taihu Lake, China. *J Hazard Mater* **2018**, 360, 43–50, doi:10.1016/j.jhazmat.2018.07.082.
9. Zhang, L.; Wang, Y.; Tan, F.; Yang, Y.; Wu, X.; Wang, W.; Liu, D. Tidal variability of polycyclic aromatic hydrocarbons and organophosphate esters in the coastal seawater of Dalian, China. *Sci Total Environ* **2019**, 134441, doi:10.1016/j.scitotenv.2019.134441.
10. Zhang, Z.; Lin, G.; Lin, T.; Zhang, R.; Jin, L.; Di, Y. Occurrence, behavior, and fate of organophosphate esters (OPEs) in subtropical paddy field environment: A case study in Nanning City of South China. *Environ Pollut* **2020**, 267, 115675, doi:10.1016/j.envpol.2020.115675.
11. Zhong, M.; Tang, J.; Guo, X.; Guo, C.; Li, F.; Wu, H. Occurrence and spatial distribution of organophosphorus flame retardants and plasticizers in the Bohai, Yellow and East China seas. *Sci Total Environ* **2020**, 741, 140434, doi:10.1016/j.scitotenv.2020.140434.
12. Zhang, R.; Yu, K.; Li, A.; Zeng, W.; Lin, T.; Wang, Y. Occurrence, phase distribution, and bioaccumulation of organophosphate esters (OPEs) in mariculture farms of the Beibu Gulf, China: A health risk assessment through seafood consumption. *Environ Pollut* **2020**, 263, 114426, doi:10.1016/j.envpol.2020.114426.

13. Dong, Z., Ma, Y., Li, J., Yuan, H., Jin, J., Wang, Y. Occurrence and Human Exposure Risk Assessment of Organophosphate Esters
14. in Drinking Water in the Weifang Binhai Economic-Technological Development Area. Environ. Chem. **2017**, 38, 4212–4219 (in Chinese).
15. Xu, L.; Hu, Q.; Liu, J.; Liu, S.; Liu, C.; Deng, Q.; Zeng, X.; Yu, Z. Occurrence of organophosphate esters and their diesters degradation products in industrial wastewater treatment plants in China: Implication for the usage and potential degradation during production processing. *Environ Pollut* **2019**, 250, 559–566, doi:10.1016/j.envpol.2019.04.058.
16. Zhang, W.; Wang, P.; Li, Y.; Wang, D.; Matsiko, J.; Yang, R.; Sun, H.; Hao, Y.; Zhang, Q.; Jiang, G. Spatial and temporal distribution of organophosphate esters in the atmosphere of the Beijing-Tianjin-Hebei region, China. *Environ Pollut* **2019**, 244, 182–189, doi:10.1016/j.envpol.2018.09.131.
17. Wang, Y., Zhang, Q., Song, N., Zhang, S., Tao, L., Zhao, Y., Han, Z. Pollution characteristics and health risk assessment of organic phosphorus flame retardant in snow water of Nanjing. China Environmental Science. **2019**, 39, 5101–5109 (in Chinese)..
18. Ren, G.; Chu, X.; Zhang, J.; Zheng, K.; Zhou, X.; Zeng, X.; Yu, Z. Organophosphate esters in the water, sediments, surface soils, and tree bark surrounding a manufacturing plant in north China. *Environ Pollut* **2019**, 246, 374–380, doi:10.1016/j.envpol.2018.12.020.
19. Luo, Q.; Gu, L.; Wu, Z.; Shan, Y.; Wang, H.; Sun, L. Distribution, source apportionment and ecological risks of organophosphate esters in surface sediments from the Liao River, Northeast China. *Chemosphere* **2020**, 250, 126297, doi:10.1016/j.chemosphere.2020.126297.
20. Cao, D.; Guo, J.; Wang, Y.; Li, Z.; Liang, K.; Corcoran, M.B.; Hosseini, S.; Bonina, S.M.C.; Rockne, K.J.; Sturchio, N.C., et al. Organophosphate Esters in Sediment of the Great Lakes. *Environ Sci Technol* **2017**, 51, 1441–1449, doi:10.1021/acs.est.6b05484.
21. Zeng, X.; Xu, L.; Hu, Q.; Liu, Y.; Hu, J.; Liao, W.; Yu, Z. Occurrence and distribution of organophosphorus flame retardants/plasticizers in coastal sediments from the Taiwan Strait in China. *Mar Pollut Bull* **2020**, 151, 110843, doi:10.1016/j.marpolbul.2019.110843.
22. Liao, C.; Kim, U.; Kannan, K. Occurrence and distribution of organophosphate esters in sediment from northern Chinese coastal waters. *Sci Total Environ* **2020**, 704, 135328, doi:10.1016/j.scitotenv.2019.135328.
23. Zeng, X.; He, L.; Cao, S.; Ma, S.; Yu, Z.; Gui, H.; Sheng, G.; Fu, J. Occurrence and distribution of organophosphate flame retardants/plasticizers in wastewater treatment plant sludges from the Pearl River Delta, China. *Environ Toxicol Chem* **2014**, 33, 1720–1725, doi:10.1002/etc.2604.
24. Xing, L.; Zhang, Q.; Sun, X.; Zhu, H.; Zhang, S.; Xu, H. Occurrence, distribution and risk assessment of organophosphate esters in surface water and sediment from a shallow freshwater Lake, China. *Sci Total Environ* **2018**, 636, 632–640, doi:10.1016/j.scitotenv.2018.04.320.
25. Pang, L.; Yang, H.; Wang, Y.; Luo, X.; Liu, S.; Xiao, J. Organophosphate flame retardants in total suspended particulates from an urban area of zhengzhou, China: Temporal variations, potential affecting factors, and health risk assessment. *Ecotox Environ Safe* **2019**, 176, 204–210, doi:10.1016/j.ecoenv.2019.03.092.
26. Ren, G.; Chen, Z.; Feng, J.; Ji, W.; Zhang, J.; Zheng, K.; Yu, Z.; Zeng, X. Organophosphate esters in total suspended particulates of an urban city in East China. *Chemosphere* **2016**, 164, 75–83, doi:10.1016/j.chemosphere.2016.08.090.
27. Chen, Y.; Song, Y.; Chen, Y.; Zhang, Y.; Li, R.; Wang, Y.; Qi, Z.; Chen, Z.; Cai, Z. Contamination profiles and potential health risks of organophosphate flame retardants in PM2.5 from Guangzhou and Taiyuan, China. *Environ Int* **2020**, 134, 105343, doi:10.1016/j.envint.2019.105343.
28. Li, J.; Tang, J.; Mi, W.; Tian, C.; Emeis, K.; Ebinghaus, R.; Xie, Z. Spatial Distribution and Seasonal Variation of Organophosphate Esters in Air above the Bohai and Yellow Seas, China. *Environ Sci Technol* **2017**, 52, 89–97, doi:10.1021/acs.est.7b03807.
29. Lai, S.; Xie, Z.; Song, T.; Tang, J.; Zhang, Y.; Mi, W.; Peng, J.; Zhao, Y.; Zou, S.; Ebinghaus, R. Occurrence and dry deposition of organophosphate esters in atmospheric particles over the northern South China Sea. *Chemosphere* **2015**, 127, 195–200, doi:10.1016/j.chemosphere.2015.02.015.
30. Cao, D.; Lv, K.; Gao, W.; Fu, J.; Wu, J.; Fu, J.; Wang, Y.; Jiang, G. Presence and human exposure assessment of organophosphate flame retardants (OPEs) in indoor dust and air in Beijing, China. *Ecotox Environ Safe* **2019**, 169, 383–391, doi:10.1016/j.ecoenv.2018.11.038.
31. Sun, Y.; Guo, J.; Liu, L.; Sverko, E.; Zhang, Z.; Tian, C.; Huo, C.; Li, H.; Ma, W.; Zhang, Z., et al. Seasonal variation and influence factors of organophosphate esters in air particulate matter of a northeastern Chinese test home. *Sci Total Environ* **2020**, 740, 140048, doi:10.1016/j.scitotenv.2020.140048.
32. Li, W.; Shi, Y.; Gao, L.; Wu, C.; Liu, J.; Cai, Y. Occurrence, distribution and risk of organophosphate esters in urban road dust in Beijing, China. *Environ Pollut* **2018**, 241, 566–575, doi:10.1016/j.envpol.2018.05.092.
33. Wang, G.; Liu, Y.; Zhao, X.; Tao, W.; Wang, H. Geographical distributions and human exposure of organophosphate esters in college library dust from Chinese cities. *Environ Pollut* **2019**, 255, 113332, doi:10.1016/j.envpol.2019.113332.
34. Pang, L.; Yang, H.; Pang, R.; Zhou, Y.; Xiao, J.; Wang, Z. Occurrence, distribution, and risk assessment of organophosphate esters in urban street dust in the central province of Henan, China. *Environ Sci Pollut Res Int* **2019**, 26, 27862–27871, doi:10.1007/s11356-019-06008-x.
35. Chen, Y.; Zhang, Q.; Luo, T.; Xing, L.; Xu, H. Occurrence, distribution and health risk assessment of organophosphate esters in outdoor dust in Nanjing, China: Urban vs. rural areas. *Chemosphere* **2019**, 231, 41–50, doi:10.1016/j.chemosphere.2019.05.135.
36. He, M.J. Organophosphate Esters in Road Dust from a Suburban Area of Chongqing, China: Characterization of Particle Size Distribution and Human Exposure. *Arch Environ Contam Toxicol* **2019**, 76, 630–639, doi:10.1007/s00244-019-00612-1.

37. Abafe, O.A.; Martincigh, B.S. Concentrations, sources and human exposure implications of organophosphate esters in indoor dust from South Africa. *Chemosphere* **2019**, 230, 239–247, doi:10.1016/j.chemosphere.2019.04.175.
38. Sun, Y.; Liu, L.Y.; Sverko, E.; Li, Y.F.; Li, H.L.; Huo, C.Y.; Ma, W.L.; Song, W.W.; Zhang, Z.F. Organophosphate flame retardants in college dormitory dust of northern Chinese cities: Occurrence, human exposure and risk assessment. *Sci Total Environ* **2019**, 665, 731–738, doi:10.1016/j.scitotenv.2019.02.098.
39. Hu, Q.; Xu, L.; Liu, Y.; Zeng, X.; Yu, Z. Co-occurrence and distribution of organophosphate tri- and di-esters in indoor dust from different indoor environments in Guangzhou and their potential human health risk. *Environ Pollut* **2020**, 262, 114311, doi:10.1016/j.envpol.2020.114311.
40. Li, W.; Wang, Y.; Kannan, K. Occurrence, distribution and human exposure to 20 organophosphate esters in air, soil, pine needles, river water, and dust samples collected around an airport in New York state, United States. *Environ Int* **2019**, 131, 105054, doi:10.1016/j.envint.2019.105054.
41. Wang, Y.; Sun, H.; Zhu, H.; Yao, Y.; Chen, H.; Ren, C.; Wu, F.; Kannan, K. Occurrence and distribution of organophosphate flame retardants (OPFRs) in soil and outdoor settled dust from a multi-waste recycling area in China. *Sci Total Environ* **2018**, 625, 1056–1064, doi:10.1016/j.scitotenv.2018.01.013.
42. He, J.; Li, J.; Ma, L.; Wu, N.; Zhang, Y.; Niu, Z. Large-scale distribution of organophosphate esters (flame retardants and plasticizers) in soil from residential area across China: Implications for current level. *Sci Total Environ* **2019**, 697, 133997, doi:10.1016/j.scitotenv.2019.133997.
43. Wan, W.; Zhang, S.; Huang, H.; Wu, T. Occurrence and distribution of organophosphorus esters in soils and wheat plants in a plastic waste treatment area in China. *Environ Pollut* **2016**, 214, 349–353, doi:10.1016/j.envpol.2016.04.038.
44. Wang, Y.; Yao, Y.; Li, W.; Zhu, H.; Wang, L.; Sun, H.; Kannan, K. A nationwide survey of 19 organophosphate esters in soils from China: Spatial distribution and hazard assessment. *Sci Total Environ* **2019**, 671, 528–535, doi:10.1016/j.scitotenv.2019.03.335.
45. He, M.; Yang, T.; Yang, Z.; Zhou, H.; Wei, S. Current State, Distribution, and Sources of Phthalate Esters and Organophosphate Esters in Soils of the Three Gorges Reservoir Region, China. *Arch Environ Con Tox* **2018**, 74, 502–513, doi:10.1007/s00244-017-0469-7.
46. Yang, Z., He, M., Yang, T., Lu, J., Wei, S. Occurrence and Distribution of the Organophosphate Esters in Soils of Mixed-land Use Area in Chongqing City. Environ. Chem. **2018**, 39, 5135–5141 (in Chinese).
47. Cui, K.; Wen, J.; Zeng, Z.; Zeng, F.; Li, S.; Zhou, X. Occurrence and distribution of organophosphate esters in urban soils of the subtropical city, Guangzhou, China. *Chemosphere* **2017**, 175, 514–520, doi:10.1016/j.chemosphere.2017.02.070.
48. Ma, Y.; Cui, K.; Zeng, F.; Wen, J.; Liu, H.; Zhu, F.; Ouyang, G.; Luan, T.; Zeng, Z. Microwave-assisted extraction combined with gel permeation chromatography and silica gel cleanup followed by gas chromatography-mass spectrometry for the determination of organophosphorus flame retardants and plasticizers in biological samples. *Anal Chim Acta* **2013**, 786, 47–53, doi:10.1016/j.aca.2013.04.062.
49. Liu, Y.; Tang, B.; Liu, Y.; Luo, X.; Mai, B.; Covaci, A.; Poma, G. Occurrence, biomagnification and maternal transfer of legacy and emerging organophosphorus flame retardants and plasticizers in water snake from an e-waste site. *Environ Int* **2019**, 133, 105240, doi:10.1016/j.envint.2019.105240.
50. Hou, R.; Liu, C.; Gao, X.; Xu, Y.; Zha, J.; Wang, Z. Accumulation and distribution of organophosphate flame retardants (PFRs) and their di-alkyl phosphates (DAPs) metabolites in different freshwater fish from locations around Beijing, China. *Environ Pollut* **2017**, 229, 548–556, doi:10.1016/j.envpol.2017.06.097.
